# Human dietary diversity in the Colombian Andes at the terminal Pleistocene-late Holocene sites Tequendama and Aguazuque

**DOI:** 10.1016/j.isci.2024.111624

**Published:** 2025-01-07

**Authors:** Michael J. Ziegler, Mark Robinson, Francisco Javier Aceituno, Gaspar Morcote-Ríos, Lorena Becerra-Valdivia, William C. Carleton, José Iriarte, Patrick Roberts

**Affiliations:** 1 isoTROPIC Research Group, Max Planck Institute of Geoanthropology, Jena, Germany; 2 Department of Archaeology, Max Planck Institute of Geoanthropology, Jena, Germany; 3 Department of Archaeology and History, University of Exeter, Exeter, UK; 4 Department of Anthropology, Faculty of Social and Human Sciences, University of Antioquia, Medellín, Colombia; 5 Instituto de Ciencias Naturales, Universidad Nacional de Colombia, Bogotá, Colombia; 6 Department of Anthropology and Archaeology, University of Bristol, Bristol, UK; 7 Linacre College, University of Oxford, Oxford, UK

**Keywords:** Paleobiology, Anthropology, Archaeology

## Abstract

Understandings of spatiotemporal dispersals of *Homo sapiens* onto the neotropical South American landscape and their environmental interactions during the late Pleistocene to late Holocene are being refined by multidisciplinary archaeological research. The Sabana of Bogota region in Colombia hosts a concentration of occupational sites, including Tequendama (13,525–2,330 and possibly until 815 cal BP) and Aguazuque (5,900–2,750 cal BP), that offer a view into local human paleoecology. Here, we conduct radiocarbon and stable isotope analysis (*δ*^13^C, *δ*^18^O and *δ*^15^N) of humans and fauna from these sites, and reveal significant interregional differences in dietary patterns through time. Specifically, individuals from Tequendama exhibit predominantly C_3_ diets, while individuals from Aguazuque show evidence of early C_4_ consumption, likely maize, around 4,400–4,200 cal BP. Stable carbon and oxygen isotope data suggest environmental stability, with periodic deviations in aridity levels within a mosaic landscape. Our study highlights the complexity of human-environment interactions in the region and contributes to a broader understanding of isotopic variability.

## Highlights

• Stable isotope data from archaeological humans and animals in Colombia
• Bayesian modeling of inter-site human occupancy with regional comparisons
• Evidence of dietary change and C_3_/C_4_ contributions from late Pleistocene to mid-Holocene
• Insights into the varying role of maize and foraging strategies in South America

## Graphical abstract

**Figure:**
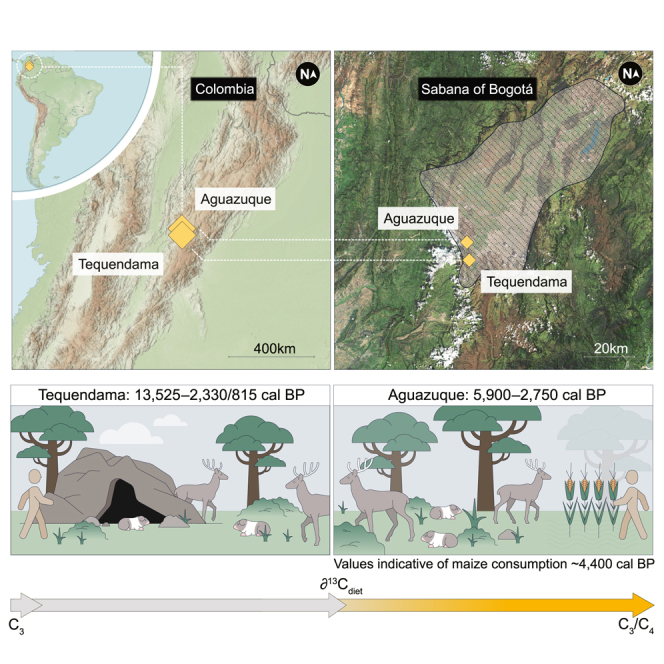

## Introduction

Research in tropical environments is crucial for understanding the adaptive flexibility of human populations dispersing during the late Pleistocene.^1^ In the South American Neotropics, *Homo sapiens* arrived between ∼20 and 12 ka when climate change during the late Pleistocene to early Holocene (LP-EH) transition was having a major impact on environments^2^ and biodiversity.^3^^,^^4^ Following arrival in the northwestern region of South America, our species would have faced a variety of diverse, and often extreme, environments including broad open savannas, seasonally-dry woodlands and forests, rugged Andean mountains, and dense, lowland Amazonian tropical forests.^5^ Mounting work from these varied South American ecosystems demonstrates LP-EH archaeological sites to be critical locations at which to refine our understanding of how early human societies interacted with neotropical flora^6^^,^^7^^,^^8^^,^^9^ and fauna.^10^^,^^11^^,^^12^ This is particularly the case given that South America is also the continent with the smallest temporal gap between initial colonization and the emergence of food production.^5^ However, how dietary patterns of early humans correspond to broader climatic and environmental changes, or reflect a reliance on particular environments (e.g., forest vs. open savanna) over time, remains relatively poorly understood for the region.

South America offers a key context to explore the tempo and nature of human adaptations to pristine environments as they were completing their colonization of the planet’s continents (other than Antarctica) by the end of the Pleistocene. There are significant debates surrounding the degree to which early human populations in South America had direct and indirect effects on endemic fauna and large-scale ecological consequences (i.e., hunting, landscape transformation, early plant cultivation).^13^^,^^14^^,^^15^^,^^16^^,^^17^^,^^18^^,^^19^^,^^20^ Studies of LP-EH human ecological adaptations in South America are becoming increasingly prominent topics in the literature, including continental overviews,^21^^,^^22^ possible depictions in early rock art^23^^,^^24^^,^^25^ associated with occupational sites,^26^ and the application of novel biomolecular approaches.^20^^,^^27^ However, there has been limited explicit discussion of regional environmental variability associated with archaeological records across northwestern South America based upon in-depth analysis of particular localities.^28^^,^^29^^,^^30^^,^^31^^,^^32^ To address this issue, site-based analysis of LP-EH localities is essential to offer direct insights into long-term human paleoecology. One such promising region is the Sabana of Bogotá in Colombia due to its geographical location, environmental diversity, and documented LP-EH archaeological contexts.

**Figure 1:**
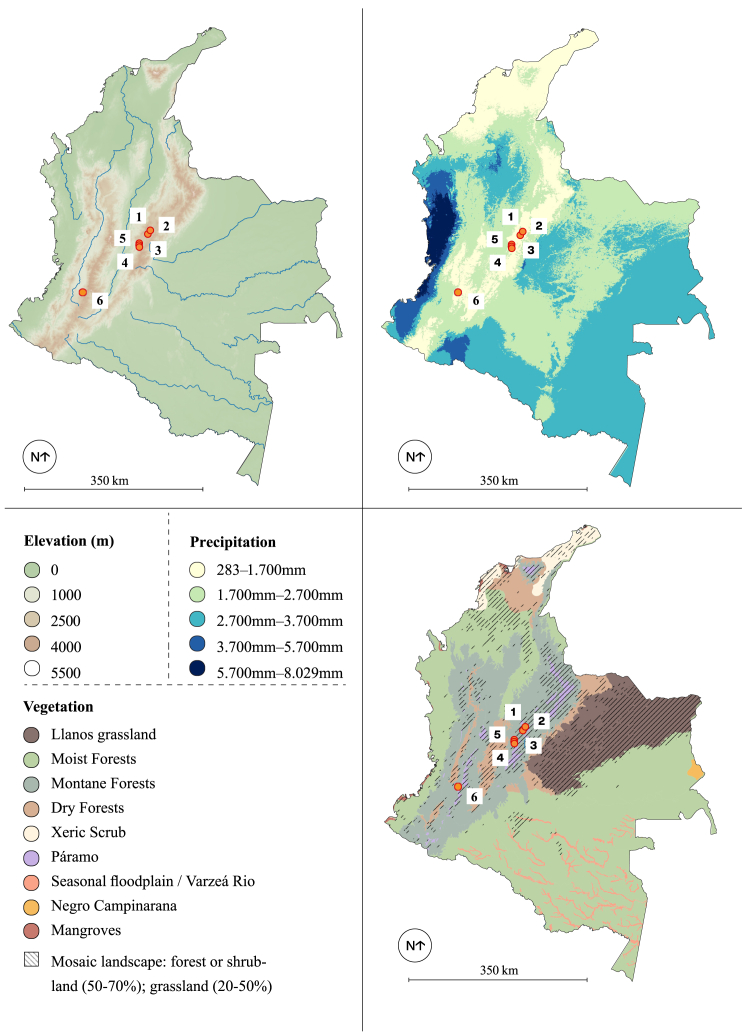
Region of interest studied Overview of Colombia with (A) elevation, (B) vegetation and (C) precipitation. LP-EH archaeological sites are represented in each map as 1) Chechua, 2) EL Abra, 3) Tequendama (4°31′59.881″ N; 74°16′30.895″ W), 4) Aguazuque (4°31′59.881″ N; 74°16′30.895″ W), 5) Nueva Esperanza and 6) San Isidro. The included scale bars represent 350 km.

Positioned in the northwestern portion of South America (Figure 1), and with some of the earliest archaeological evidence of human presence on the continent, this region would have been a particularly important corridor, facilitating the dispersal of *Homo sapiens* into South American landscapes.^29^ Paleoecological,^33^ zooarchaeological,^34^ and archaeobotanical^35^^,^^36^ studies suggest distinct periods of climatic and environmental variability impacting mosaic savanna and high-altitude forest environments across the LP-EH. This includes dramatic shifts in regional forestscapes (i.e., moist, montane, dry) and Páramo grassland boundaries,^37^ including periods of compositional changes (i.e., C_3_ and C_4_ plant distributions; Methods S1). Archaeological studies in the Bogotá region have managed to partially reconstruct subsistence of pre-agricultural societies in Colombia,^38^ with major contributions from historical excavations at LP-EH localities like Tequendama (13,525–2,330 and possibly until 815 cal BP; 2,570 m a.s.l.)^39^ and Aguazuque (5,900–2,750 cal BP; 2,550 m a.s.l.).^40^ Nevertheless, comprehensive insights into human subsistence strategies by further direct analyses of archaeological material from these sites have remained somewhat lacking. We seek to further investigate the nature and duration of human occupation at these sites. We speculate that the application of biomolecular analyses will reveal refined temporal and geographic variation of early human diets throughout and between the Tequendama and Aguazuque occupational sequences based on their different ecological and cultural settings.

Here, we apply radiocarbon dating and Bayesian modeling techniques to test the fidelity of previous site chronologies and provide a more nuanced understanding of human occupancy at Tequendama and Aguazuque. Additionally, we apply stable carbon, oxygen and nitrogen isotope analysis to human and animal remains from these sites’ archaeological contexts to investigate reliance on different resources.^41^^,^^42^ In Neotropical contexts, this methodology can be used to explore human reliance on forest biomes (dominated by C_3_ vegetation) versus grassland biomes (dominated by C_4_ vegetation).^13^^,^^27^^,^^43^^,^^44^^,^^45^ Within C_3_-dominated ecosystems, stable carbon isotope values are impacted by the ‘canopy effect’, providing further insights into forest habitats and densities as well as tropical adaptations.^46^^,^^47^ Furthermore, stable oxygen isotope ratios of human and animal remains can provide insights into hydrological conditions and drinking behaviors of analyzed taxa.^48^ In this study, we undertake the most extensive isotopic analysis of LP-EH localities in the Bogotá region based on archaeological samples from Tequendama and Aguazuque. Specifically, we seek to explore the degree to which human diets varied between the two sites, comparing *δ*^13^C from tooth enamel and postcranial bone collagen to study dietary inputs. This includes an assessment of the degree of human reliance on neotropical forest versus more open settings through time and the relationship of these observations to climatic changes. We aim to create a high-fidelity record of the environmental and dietary history of LP-EH humans and fauna from these critical sites, in an important region of South America, to explore the dynamic adaptations of our species on their continental ‘Last Journey’.

## Results

**Figure 2:**
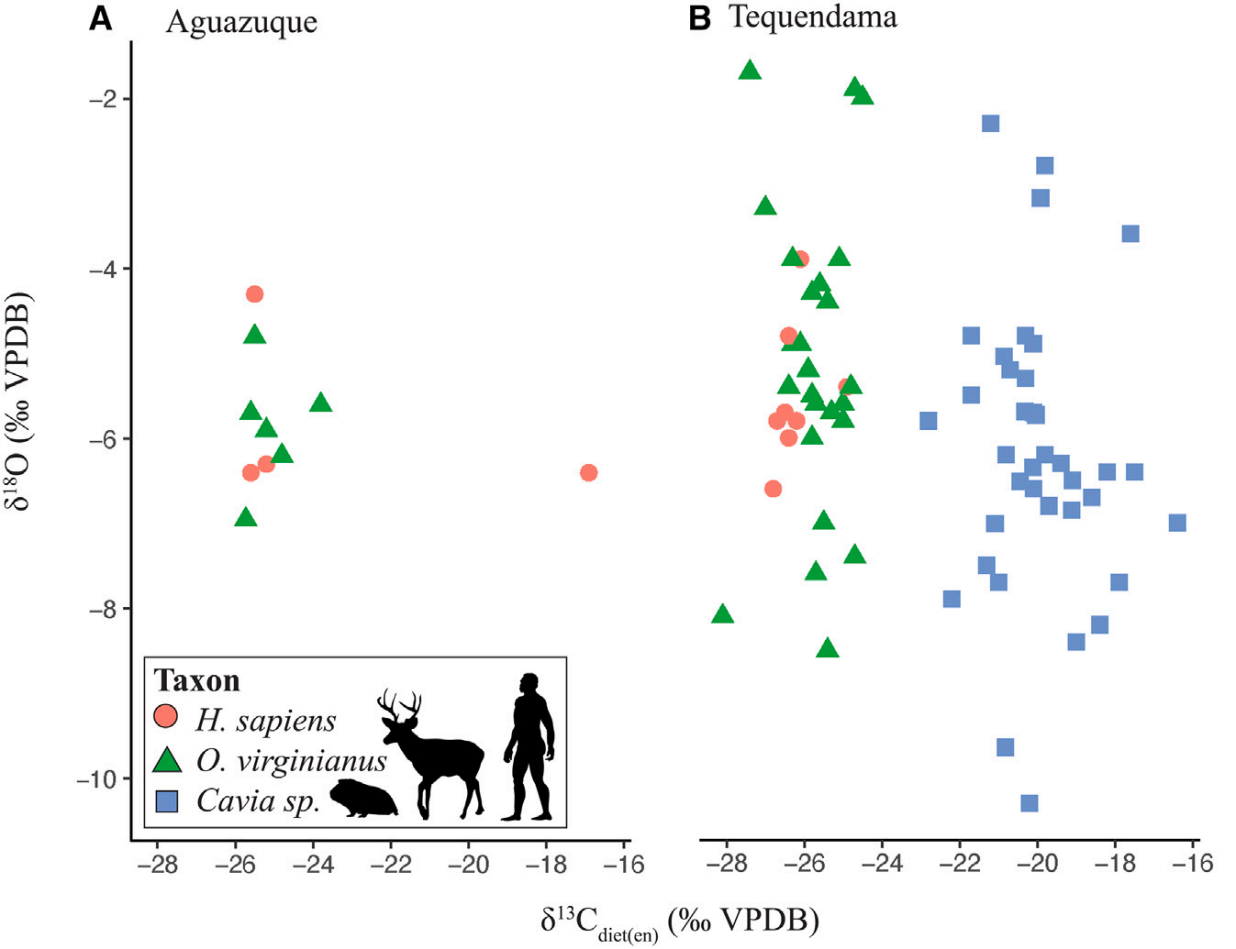
Archaeological site data Isotopic overview of δC_diet(en)_ and δ^18^O stable isotope data for (A) Aguazuque and (B) Tequendama.

**Figure 3:**
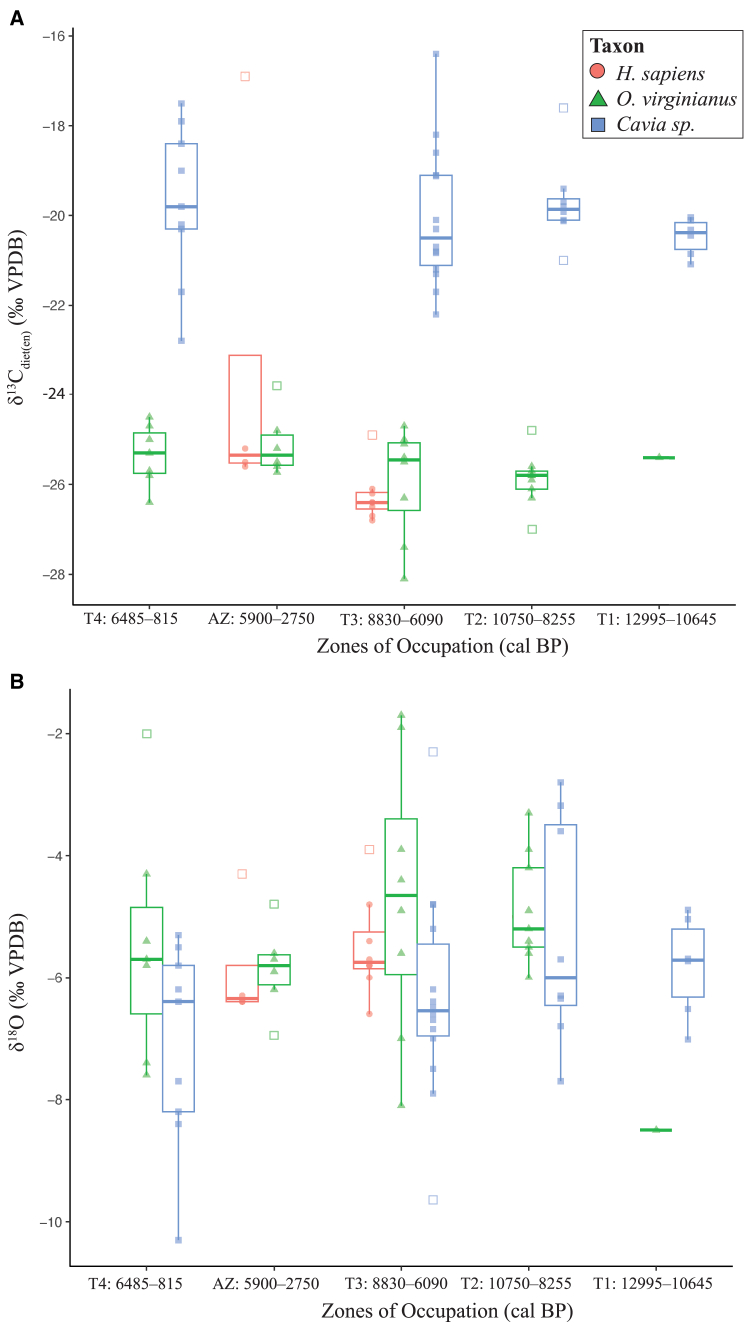
Temporal comparison of archaeological sites Isotopic overview of (A) δ^13^C_diet(en)_ and (B) δ^18^O stable isotope data from Tequendama and Aguazuque by zone of occupation.

**Figure 4:**
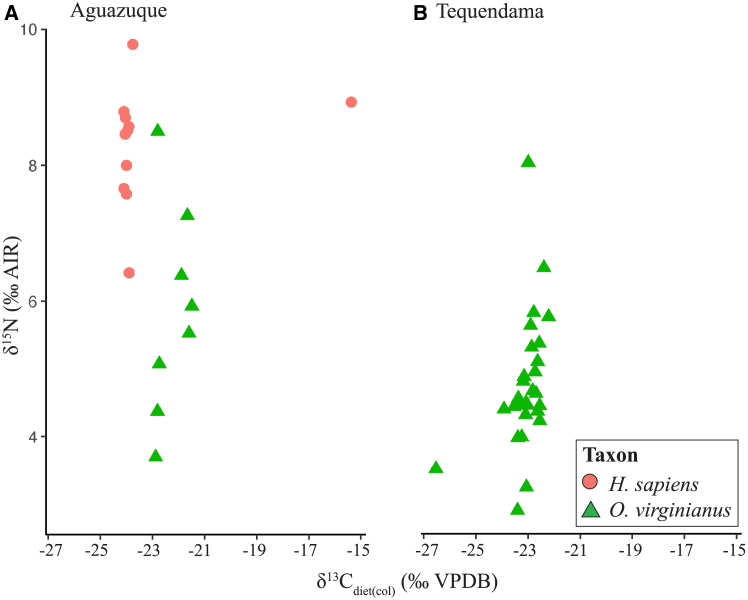
Archaeological site data Isotopic overview of δ^13^C_diet(col)_ and δ15N from bone collagen at (A) Aguazuque and (B) Tequendama.

The full stable isotope datasets of *δ*^13^C_(raw)_, *δ*^13^C_diet(en)_, *δ*^18^O, *δ*^13^C_diet(col)_ and *δ*^15^N measurements for humans and selected fauna from Tequendama and Aguazuque are shown in Table 1 and Figure 2. Our data provide 127 data points, including *δ*C_diet(en)_ and *δ*^18^O (*n* = 80) data as well as *δ*^13^C_diet(col)_ and *δ*^15^N (*n* = 47) data across both sites from enamel and collagen, respectively. An enrichment factor of +9.9‰ for *Cavia* sp.,^49^ +14.0‰ for *H. sapiens* and +14.5‰ for *Odocoileus virginianus*^50^ was applied to raw *δ*^13^C values from tooth enamel bioapatite in order to compare diet between the varied species that process food differently,^51^ resulting in a taxonomically comparable *δ*C_diet(en)_ value (see STAR methods section). All subsequent usage of *δ*^18^O refers to oxygen isotope values from the carbonate fraction of enamel bioapatite. For *δ*^13^C values from bone collagen, a discrimination factor of +3‰–4*‰**^52^*^*,*^^53^ was considered in order to produce a *δ*^13^C_diet(col)_ value and better interpret dietary contributions. All usage of *δ*^15^N refers to nitrogen isotope values from bone collagen and an interpreted trophic enrichment factor of +3‰–5*‰* was assumed (see STAR Methods section). We studied site-specific results of *δ*C_diet(en)_ and *δ*^18^O data at Tequendama and Aguazuque (Figure 2), exploring data by their zones of occupation (Figure S1) which represent chronologically distinct time bins over the LP-EH (Table 1). Furthermore, spatiotemporal comparisons of *δ*C_diet(en)_ and *δ*^18^O isotopic data across all included taxa from both Tequendama and Aguazuque were also undertaken (Figure 3). This taxonomic comparison is also conducted for *δ*^13^C and *δ*^15^N data from bone collagen across both sites (Figure 4).

**Table 1:** Stable isotope measurements of bulk enamel (δC_diet(en)_, δ^18^O) and bone collagen (δ^13^C_diet(col)_, δ^15^N) material from human and faunal specimens sampled through the archaeological contexts of Tequendama and Aguazuque

Site/Zone	Age (cal BP)	ID	Taxon	Tooth/Bone	*δ*^13^C_raw_	*δ*^13^C_diet(col)_	*δ*^13^C(σ)	*δ*^15^N	*δ*^15^N(σ)	C:N	*δ*^13^C_diet(en)_	*δ*^13^C(σ)	*δ*^18^O	*δ*^18^O(σ)
Teq. (T4)	6485–815	T03-02	*O. virginianus*	m3(R)	−12,39	–	–	–	–	–	−26,89	0,3	−5,41	0,3
Teq. (T4)	6485–815	T09-02	*O. virginianus*	p3(L)	−10,53	–	–	–	–	–	−25,03	0,3	−2,04	0,2
Teq. (T4)	6485–815	T15-01	*O. virginianus*	M3(L)	−10,66	–	–	–	–	–	−25,16	0,3	−7,41	0,3
Teq. (T4)	6485–815	T16-01	*O. virginianus*	M3(R)	−11,82	–	–	–	–	–	−26,32	0,3	−4,31	0,3
Teq. (T4)	6485–815	T16-02	*O. virginianus*	M2(R)	−11,69	–	–	–	–	–	−26,19	0,3	−7,56	0,3
Teq. (T4)	6485–815	T18-01	*O. virginianus*	m3(R)	−11,32	–	–	–	–	–	−25,82	0,3	−5,65	0,3
Teq. (T4)	6485–815	T20-01	*O. virginianus*	p3(L)	−10,96	–	–	–	–	–	−25,46	0,3	−5,80	0,1
Teq. (T4)	6485–815	T01-01	*O. virginianus*	Scapula (R)	−19,55	−22,55	0,0	4,45	0,0	3,2	–	–	–	–
Teq. (T4)	6485–815	T01-02	*O. virginianus*	Radius (R)	−19,73	−22,73	0,1	4,95	0,0	3,1	–	–	–	–
Teq. (T4)	6485–815	T04-02	*O. virginianus*	Os Coxae (R)	−19,57	−22,57	0,1	5,37	0,0	3,2	–	–	–	–
Teq. (T4)	6485–815	T04-03	*O. virginianus*	Rib	−23,53	−26,53	0,2	3,52	0,0	3,2	–	–	–	–
Teq. (T4)	6485–815	T04-04	*O. virginianus*	Tibia (L)	−20,39	−23,39	0,0	3,98	0,1	3,3	–	–	–	–
Teq. (T4)	6485–815	T06-02	*O. virginianus*	Femur (L)	−20,06	−23,06	0,1	3,25	0,1	3,1	–	–	–	–
Teq. (T4)	6485–815	T06-04	*O. virginianus*	Sacrum	−20,18	−23,18	0,0	4,81	0,2	3,1	–	–	–	–
Teq. (T4)	6485–815	T09-05	*O. virginianus*	Vertebra	−19,90	−22,90	0,0	5,63	0,2	3,2	–	–	–	–
Teq. (T4)	6485–815	T15-04	*O. virginianus*	Scapula (R)	−20,92	−23,92	0,1	4,40	0,3	3,2	–	–	–	–
Teq. (T4)	6485–815	T16-03	*O. virginianus*	Tibia (L)	−20,09	−23,09	0,3	4,32	0,1	3,3	–	–	–	–
Teq. (T4)	6485–815	T16-05	*O. virginianus*	Radius (R)	−20,24	−23,24	0,3	3,99	0,1	3,1	–	–	–	–
Teq. (T4)	6485–815	T16-06	*O. virginianus*	Ulna (R)	−19,78	−22,78	0,0	5,82	0,0	3,2	–	–	–	–
Teq. (T4)	6485–815	T18-03	*O. virginianus*	Thoracic vert.	−19,69	−22,69	0,0	4,63	0,0	3,1	–	–	–	–
Teq. (T4)	6485–815	T18-04	*O. virginianus*	Rib	−19,55	−22,55	0,1	4,23	0,1	3,2	–	–	–	–
Teq. (T4)	6485–815	T18-05	*O. virginianus*	Humerus (L)	−19,82	−22,82	0,1	4,67	0,1	3,2	–	–	–	–
Teq. (T4)	6485–815	T10-01	*Cavia* sp.	incisor	−7,62	–	–	–	–	–	−17,52	0,2	−6,42	0,1
Teq. (T4)	6485–815	T10-04	*Cavia* sp.	incisor	−7,99	–	–	–	–	–	−17,89	0,2	−7,72	0,1
Teq. (T4)	6485–815	T11-01	Cavia sp.	incisor	−10,29	–	–	–	–	–	−20,19	0,2	−10,35	0,2
Teq. (T4)	6485–815	T11-02	*Cavia* sp.	incisor	−10,42	–	–	–	–	–	−20,32	0,3	−5,32	0,2
Teq. (T4)	6485–815	T12-01	*Cavia* sp.	incisor	−9,06	–	–	–	–	–	−18,96	0,2	−8,37	0,2
Teq. (T4)	6485–815	T13-02	*Cavia* sp.	incisor	−8,47	–	–	–	–	–	−18,37	0,2	−8,22	0,2
Teq. (T4)	6485–815	T14-02	*Cavia* sp.	incisor	−9,89	–	–	–	–	–	−19,79	0,2	−6,15	0,2
Teq. (T4)	6485–815	T17-02	*Cavia* sp.	incisor	−11,80	–	–	–	–	–	−21,70	0,3	−5,51	0,2
Teq. (T4)	6485–815	T17-03	*Cavia* sp.	incisor	−12,89	–	–	–	–	–	−22,79	0,2	−5,81	0,1
Teq. (T3)	8830–6090	T21-01	*O. virginianus*	p3(R)	−10,67	–	–	–	–	–	−25,17	0,3	−1,90	0,2
Teq. (T3)	8830–6090	T23-03	*O. virginianus*	m2(L)	−14,10	–	–	–	–	–	−28,60	0,3	−8,06	0,2
Teq. (T3)	8830–6090	T25-01	*O. virginianus*	m3(L)	−11,53	–	–	–	–	–	−26,03	0,2	−7,04	0,2
Teq. (T3)	8830–6090	T27-01	*O. virginianus*	M3(R)	−11,38	–	–	–	–	–	−25,88	0,3	−4,37	0,2
Teq. (T3)	8830–6090	T30-02	*O. virginianus*	M3(L)	−11,10	–	–	–	–	–	−25,60	0,3	−3,93	0,2
Teq. (T3)	8830–6090	T32-02	*O. virginianus*	p3(L)	−12,31	–	–	–	–	–	−26,81	0,3	−4,86	0,2
Teq. (T3)	8830–6090	T35-01	*O. virginianus*	M3(L)	−13,36	–	–	–	–	–	−27,86	0,3	−1,73	0,2
Teq. (T3)	8830–6090	T35-02	*O. virginianus*	M2(L)	−11,01	–	–	–	–	–	−25,51	0,2	−5,56	0,2
Teq. (T3)	8830–6090	T21-06	*O. virginianus*	Rib	−19,86	−22,86	0,0	5,31	0,0	3,2	–	–	–	–
Teq. (T3)	8830–6090	T27-02	*O. virginianus*	Os Coxae (R)	−20,28	−23,28	0,3	4,48	0,1	3,2	–	–	–	–
Teq. (T3)	8830–6090	T27-03	*O. virginianus*	Os Coxae (R)	−20,53	−23,53	0,2	4,44	0,2	3,3	–	–	–	–
Teq. (T3)	8830–6090	T27-04	*O. virginianus*	Humerus (L)	−20,08	−23,08	0,0	4,49	0,0	3,3	–	–	–	–
Teq. (T3)	8830–6090	T30-03	*O. virginianus*	Humerus (L)	−19,99	−22,99	0,0	8,03	0,0	3,1	–	–	–	–
Teq. (T3)	8830–6090	T30-04	*O. virginianus*	Tibia (R)	−19,39	−22,39	0,1	6,48	0,1	3,2	–	–	–	–
Teq. (T3)	8830–6090	T30-05	*O. virginianus*	Ulna (R)	−20,02	−23,02	0,0	4,46	0,1	3,1	–	–	–	–
Teq. (T3)	8830–6090	T34-03	*O. virginianus*	Femur (R)	−19,63	−22,63	0,3	4,37	0,0	3,1	–	–	–	–
Teq. (T3)	8830–6090	T22-01	*Cavia* sp.	incisor	−8,27	–	–	–	–	–	−18,17	0,2	−6,36	0,2
Teq. (T3)	8830–6090	T22-04	*Cavia* sp.	incisor	−10,83	–	–	–	–	–	−20,73	0,3	−5,23	0,2
Teq. (T3)	8830–6090	T24-01	*Cavia* sp.	incisor	−6,47	–	–	–	–	–	−16,37	0,3	−7,04	0,2
Teq. (T3)	8830–6090	T24-02	*Cavia* sp.	incisor	−8,72	–	–	–	–	–	−18,62	0,3	−6,69	0,2
Teq. (T3)	8830–6090	T28-02	*Cavia* sp.	incisor	−11,77	–	–	–	–	–	−21,67	0,3	−4,77	0,2
Teq. (T3)	8830–6090	T28-03	*Cavia* sp.	incisor	−10,22	–	–	–	–	–	−20,12	0,2	−6,57	0,1
Teq. (T3)	8830–6090	T29-01	*Cavia* sp.	incisor	−9,22	–	–	–	–	–	−19,12	0,2	−6,54	0,2
Teq. (T3)	8830–6090	T29-03	*Cavia* sp.	incisor	−12,30	–	–	–	–	–	−22,20	0,2	−7,87	0,1
Teq. (T3)	8830–6090	T31-03	*Cavia* sp.	incisor	−11,45	–	–	–	–	–	−21,35	0,1	−7,53	0,1
Teq. (T3)	8830–6090	T33-01	*Cavia* sp.	incisor	−11,28	–	–	–	–	–	−21,18	0,2	−2,27	0,2
Teq. (T3)	8830–6090	T33-02	*Cavia* sp.	incisor	−10,91	–	–	–	–	–	−20,81	0,3	−6,18	0,2
Teq. (T3)	8830–6090	T36-02	*Cavia* sp.	incisor	−10,36	–	–	–	–	–	−20,26	0,2	−4,76	0,2
Teq. (T3)	8830–6090	T26-04	*Cavia* sp.	incisor	−10,93	–	–	–	–	–	−20,83	0,3	−9,64	0,2
Teq. (T3)	8830–6090	T26-05	*Cavia* sp.	incisor	−9,22	–	–	–	–	–	−19,12	0,3	−6,85	0,3
Teq. (T3)	8830–6090	T01	*Homo sapiens*	M2(R)	−12,71	–	–	–	–	–	−26,71	0,3	−5,89	0,2
Teq. (T3)	8830–6090	T03	*Homo sapiens*	m2(R)	−12,09	–	–	–	–	–	−26,09	0,2	−3,93	0,3
Teq. (T3)	8830–6090	T04	*Homo sapiens*	M2(R)	−12,39	–	–	–	–	–	−26,39	0,4	−4,83	0,3
Teq. (T3)	8830–6090	T05	*Homo sapiens*	M3(R)	−12,22	–	–	–	–	–	−26,22	0,2	−5,83	0,1
Teq. (T3)	8830–6090	T06	*Homo sapiens*	M2(L)	−12,85	–	–	–	–	–	−26,85	0,2	−6,62	0,1
Teq. (T3)	8830–6090	T07	*Homo sapiens*	M3(R)	−10,85	–	–	–	–	–	−24,85	0,2	−5,35	0,6
Teq. (T3)	8830–6090	T08a	*Homo sapiens*	m2(R)	−12,65	–	–	–	–	–	−26,65	0,2	−6,17	0,1
Teq. (T3)	8830–6090	T08b	*Homo sapiens*	M2(L)	−12,73	–	–	–	–	–	−26,73	0,3	−5,78	0,2
Teq. (T2)	10750–8255	T37-01	*O. virginianus*	p3(R)	−11,70	–	–	–	–	–	−26,20	0,3	−5,60	0,2
Teq. (T2)	10750–8255	T38-01	*O. virginianus*	M3(R)	−11,80	–	–	–	–	–	−26,30	0,2	−5,50	0,2
Teq. (T2)	10750–8255	T42-01	*O. virginianus*	m3(R)	−12,99	–	–	–	–	–	−27,49	0,3	−3,26	0,2
Teq. (T2)	10750–8255	T43-01	*O. virginianus*	p3(R)	−10,82	–	–	–	–	–	−25,32	0,2	−5,35	0,2
Teq. (T2)	10750–8255	T45-01	*O. virginianus*	m2(L)	−11,83	–	–	–	–	–	−26,33	0,3	−5,98	0,2
Teq. (T2)	10750–8255	T46-01	*O. virginianus*	m3(R)	−11,62	–	–	–	–	–	−26,12	0,2	−4,22	0,2
Teq. (T2)	10750–8255	T48-01	*O. virginianus*	p3(R)	−12,32	–	–	–	–	–	−26,82	0,2	−3,87	0,2
Teq. (T2)	10750–8255	T48-02	*O. virginianus*	m2(R)	−11,95	–	–	–	–	–	−26,45	0,3	−5,25	0,2
Teq. (T2)	10750–8255	T51-01	*O. virginianus*	m3(R)	−12,05	–	–	–	–	–	−26,55	0,3	−4,93	0,2
Teq. (T2)	10750–8255	T40-01	*O. virginianus*	Astragalus (L)	−20,40	−23,40	0,3	2,91	0,1	3,2	–	–	–	–
Teq. (T2)	10750–8255	T42-04	*O. virginianus*	Metacarpal (L)	−19,21	−22,21	0,2	5,76	0,1	3,3	–	–	–	–
Teq. (T2)	10750–8255	T43-04	*O. virginianus*	Long Bone	−20,37	−23,37	0,3	4,56	0,2	3,3	–	–	–	–
Teq. (T2)	10750–8255	T39-01	*Cavia* sp.	incisor	−7,72	–	–	–	–	–	−17,62	0,2	−3,64	0,2
Teq. (T2)	10750–8255	T39-02	*Cavia* sp.	incisor	−11,10	–	–	–	–	–	−21,00	0,3	−7,67	0,4
Teq. (T2)	10750–8255	T41-01	*Cavia* sp.	incisor	−9,51	–	–	–	–	–	−19,41	0,3	−6,29	0,3
Teq. (T2)	10750–8255	T44-01	*Cavia* sp.	incisor	−10,21	–	–	–	–	–	−20,11	0,2	−5,69	0,1
Teq. (T2)	10750–8255	T49-01	*Cavia* sp.	incisor	−9,77	–	–	–	–	–	−19,67	0,3	−6,79	0,3
Teq. (T2)	10750–8255	T52-01	*Cavia* sp.	incisor	−9,87	–	–	–	–	–	−19,77	0,2	−2,84	0,1
Teq. (T2)	10750–8255	T47-02	*Cavia* sp.	incisor	−10,22	–	–	–	–	–	−20,12	0,3	−6,35	0,3
Teq. (T2)	10750–8255	T52-01	*Cavia* sp.	incisor	−10,02	–	–	–	–	–	−19,92	0,2	−3,18	0,1
Teq. (T1)	12995–10645	T65-01	*O. virginianus*	p3(L)	−11,45	–	–	–	–	–	−25,95	0,4	−8,51	0,3
Teq. (T1)	12995–10645	T56-06	*O. virginianus*	Rib	−20,15	−23,15	0,0	4,88	0,2	3,2	–	–	–	–
Teq. (T1)	12995–10645	T66-01	*O. virginianus*	Cervical vert.	−19,63	−22,63	0,3	5,10	0,3	3,4	–	–	–	–
Teq. (T1)	12995–10645	T60-01	*Cavia* sp.	incisor	−10,21	–	–	–	–	–	−20,11	0,3	−4,89	0,3
Teq. (T1)	12995–10645	T60-02	*Cavia* sp.	incisor	−10,96	–	–	–	–	–	−20,86	0,2	−5,04	0,1
Teq. (T1)	12995–10645	T67-01	*Cavia* sp.	incisor	−10,55	–	–	–	–	–	−20,45	0,2	−6,51	0,1
Teq. (T1)	12995–10645	T67-02	*Cavia* sp.	incisor	−10,14	–	–	–	–	–	−20,04	0,3	−5,73	0,3
Teq. (T1)	12995–10645	T68-01	*Cavia* sp.	incisor	−10,42	–	–	–	–	–	−20,32	0,2	−5,69	0,1
Teq. (T1)	12995–10645	T68-02	*Cavia* sp.	incisor	−11,19	–	–	–	–	–	−21,09	0,2	−7,01	0,1
Agz. (A5)	2900–2750	A25-01	*Homo sapiens*	M3(R)	−11,59	–	–	–	–	–	−25,59	0,3	−6,43	0,2
Agz. (A5)	2900–2750	A28-01	*O. virginianus*	m3(L)	−10,84	–	–	–	–	–	−25,34	0,3	−6,25	0,2
Agz. (A5)	2900–2750	A29-01	*O. virginianus*	m3(L)	−11,73	–	–	–	–	–	−26,23	0,2	−6,95	0,1
Agz. (A5)	2900–2750	A26-01	*Homo sapiens*	Tibia (R)	−20,10	−24,10	0,2	8,79	0,1	3,3	–	–	–	–
Agz. (A5)	2900–2750	A27-01	*Homo sapiens*	Radius (L)	−11,37	−15,37	0,0	8,93	0,1	3,2	–	–	–	–
Agz. (A5)	2900–2750	A30-01	*O. virginianus*	Rib	−19,80	−22,80	0,1	8,50	0,0	3,2	–	–	–	–
Agz. (A5)	2900–2750	A31-01	*O. virginianus*	Rib	−19,74	−22,74	0,3	5,08	0,1	3,2	–	–	–	–
Agz. (A4)	3750–3550	A23-01	*Homo sapiens*	Tibia (L)	−20,00	−24,00	0,1	8,00	0,1	3,2	–	–	–	–
Agz. (A4)	3750–3550	A24-01	*Homo sapiens*	Scapula (R)	−19,91	−23,91	0,1	8,57	0,2	3,2	–	–	–	–
Agz. (A3)	4400–4200	A15-01	*Homo sapiens*	m3(R)	−11,20	–	–	–	–	–	−25,20	0,3	−6,32	0,1
Agz. (A3)	4400–4200	A19-01	*O. virginianus*	m3(R)	−9,80	–	–	–	–	–	−24,30	0,3	−5,62	0,2
Agz. (A3)	4400–4200	A16-01	*Homo sapiens*	Femur (R)	−20,05	−24,05	0,1	8,46	0,1	3,3	–	–	–	–
Agz. (A3)	4400–4200	A17-01	*Homo sapiens*	Tibia (L)	−20,04	−24,04	0,0	8,70	0,1	3,2	–	–	–	–
Agz. (A3)	4400–4200	A18-01	*Homo sapiens*	Humerus (R)	−19,76	−23,76	0,1	9,78	0,1	3,2	–	–	–	–
Agz. (A3)	4400–4200	A20-01	*O. virginianus*	Tibia (L)	−18,89	−21,89	0,0	6,38	0,0	3,2	–	–	–	–
Agz. (A3)	4400–4200	A21-01	*O. virginianus*	Tibia (R)	−18,49	−21,49	0,0	5,93	0,1	3,2	–	–	–	–
Agz. (A3)	4400–4200	A22-01	*O. virginianus*	Long bone	−18,66	−21,66	0,1	7,26	0,2	3,2	–	–	–	–
Agz. (A2)	4400–4200	A08-01	*Homo sapiens*	m3(L)	−2,91	–	–	–	–	–	−16,91	0,3	−6,43	0,2
Agz. (A2)	4400–4200	A12-01	*O. virginianus*	m3(R)	−11,54	–	–	–	–	–	−26,04	0,3	−4,77	0,2
Agz. (A2)	4400–4200	A09-01	*Homo sapiens*	Femur (R)	−20,10	−24,10	0,1	7,66	0,1	3,2	–	–	–	–
Agz. (A2)	4400–4200	A11-01	*Homo sapiens*	Radius (L)	−19,95	−23,95	0,1	8,52	0,1	3,2	–	–	–	–
Agz. (A2)	4400–4200	A13-01	*O. virginianus*	Rib	−19,88	−22,88	0,1	3,70	0,0	3,2	–	–	–	–
Agz. (A2)	4400–4200	A14-01	*O. virginianus*	Rib	−19,81	−22,81	0,0	4,37	0,1	3,2	–	–	–	–
Agz. (A1)	5900–5650	A01-01	*Homo sapiens*	M2(R)	−11,53	–	–	–	–	–	−25,53	0,3	−4,33	0,2
Agz. (A1)	5900–5650	A04-01	*O. virginianus*	m3(L)	−11,18	–	–	–	–	–	−25,68	0,3	−5,89	0,1
Agz. (A1)	5900–5650	A05-01	*O. virginianus*	m3(R)	−11,55	–	–	–	–	–	−26,05	0,3	−5,73	0,2
Agz. (A1)	5900–5650	A01-01	*Homo sapiens*	Tibia (R)	−19,86	−23,9	0,0	6,42	0,0	3,1	–	–	–	–
Agz. (A1)	5900–5650	A03-01	*Homo sapiens*	Radius (R)	−19,96	−24,0	0,0	7,58	0,0	3,2	–	–	–	–
Agz. (A1)	5900–5650	A07-01	*O. virginianus*	Metatarsal (R)	−18,61	−21,6	0,1	5,53	0,1	3,2	–	–	–	–

All isotopic values (i.e., *δ*^13^C, *δ*^15^N and *δ*^18^O) are reported in units per mil (‰). Resulting *δ*^13^C and *δ*^18^O from enamel as well as *δ*^13^C from bone collagen were calibrated relative to the VPDB; *δ*^15^N from bone collagen calibrated relative to the AIR. Considering human and faunal bone collagen *δ*^13^C is expected to be higher than in the diet, we applied +3‰–4‰ as a discrimination factor to our *δ*^13^C_raw_ values from bone colllagen in order to produce *δ*^13^C_diet(col)_ and better interpret contribution of C3 and C4 plant material in diet. Similarly, in *δ*^13^C_raw_ values from enamel, an enrichment factor of +14‰ is used for human^54^^,^^55^^,^^56^^,^^57^ and +14.5‰ for deer^50^ whereas, a +9.9‰ enrichment factor is used for guniea pig^49^^,^^58^^,^^59^^,^^60^ to produce *δ*^13^C_diet(en)_.

### Tequendama sequence: Radiocarbon dates and chronology

We present radiocarbon dates (*n* = 6) from throughout the Tequendama sequence and its previously-established zones of occupation (T1–T4). These dates are detailed in Table 2 and build upon the chronology of the site surmised from previous publications (Methods S2). The incorporation of calibrated radiocarbon dates from this study into a composite dataset or previously published dates permits the evaluation of temporal zones by Bayesian analyses (Figures S5 and S6). Overall, results from these analyses generally support the zones of occupation originally described at Tequendama^39^ and estimate a refined site chronology. Specifically, Bayesian models estimate that the occupational history has boundaries ranging 13,525–2330. In our model, the Boundary Function estimates this older commencement of the Tequendama sequence, however we use the following Phase Function results to better define estimated zones of occupation; T1 (12,995–10,645 cal BP), T2 (10,750–8,255 cal BP), T3 (8,830–6,090 cal BP) and T4 (6,485–815 cal BP).

**Table 2:** Archaeological and chronometric data from the Tequendama sequence

Site	Lab ID	Sample ID	Taxa	Material; Element	Zone	Strata; Grid; z	ORAU Pcode	Collagen Yield (%)	C:N	C (%)	N (%)	δ^13^C	δ^15^N	Date (BP)	±	Date (cal BP; 95.4% CI)
Teq. I	OxA-43251	DA-TEQ1121 56-03	*O. virginianus*	Bone; Metatarsal	T1	5b; D1; 1.9	AG	2.1	3.4	41.1	14.3	−19.4	4.1	9490	32	11070–10585
Teq. I	OxA-43250	DA-TEQ1121 56-03	*O. virginianus*	Bone; Metatarsal	T1	5b; D1; 1.9	AF	∗	3.3	44.6	15.7	−19.5	4.5	9487	31	11070–10585
Teq. I	OxA-43307	DA-TEQ1121 37-03	*O. virginianus*	Bone; Vertebrae	T2	7a/7b; D1; 1.5	AF	4.1	3.3	44.3	15.8	−19.7	5.1	9287	29	10580–10305
Teq. I	OxA-43306	DA-TEQ1121 42-02	*O. virginianus*	Bone; Metacarpal	T2	7a/7b; A4; 2.4	AF	3.9	3.3	44.0	15.6	−21.1	4.4	9026	28	10240–10175
Teq. I	OxA-43304	DA-TEQ1121 18-02	*O. virginianus*	Bone; Calcaneus	T3	8; A2; 0.3	AG	13.0	3.2	43.0	15.6	−20.1	4.6	5773	22	6650–6495
Teq. I	OxA-43305	DA-TEQ1121 34-01	*O. virginianus*	Bone; Scapula	T4	8/9; M; 1.1	AF	7.0	3.1	42.8	15.9	−20.6	3.5	5081	20	5910–5745

Samples OxA-43250 and 43251 are duplicates. The percent collagen is the yield of extracted collagen as a function of the starting weight of bone samples. Denoted with an asterisks, ∗AG collagen was ultrafiltered, so there is no collagen yield value to report for OxA-43250. C:N is the atomic weight ratio of carbon to nitrogen. %C and % N is the percentage of carbon and nitrogen in the combusted sample. z refers to depth in meters. BP refers to 'before present', with present for radiocarbon measurements set at AD 1950. Date error (±) is to 1σ. Calibrated ages (cal BP) were produced using the IntCal20 curve.^61^ CI refers to confidence interval.

### Tequendama sequence: δC_diet(en)_ and δ^18^O from enamel

Representative fauna, including white-tailed deer (*O. virginianus*) and guinea pig (*Cavia* sp.), from Tequendama were found in abundance across all zones of occupation. Enamel samples of *O. virginianus* (*n* = 25) exhibit *δ*C_diet(en)_ values from −28.6 to −25.0‰ and a mean of −26.2 ± 0.3‰, whereas guinea pig (*n* = 37) *δ*C_diet(en)_ values from −22.8 to −16.4‰ and a mean of −20.0 ± 0.2‰ have a wider range and higher overall values. *δ*^18^O data differs taxonomically where the majority of *Cavia* sp. values (range = −10.4 to −2.3‰; average = −6.2 ± 0.2‰) are lower than their O. *virginianus* counterpart (range = −8.5 to −1.7‰; average = −5.1 ± 0.2‰), with the exception of zone T1 (12,995–10,645 cal BP). Corresponding statistical Mann-Whitney-U (W = 925; *p* = <0.05) and a *t*-test (*t* = −0.50276, *p* = 0.64) both suggest significant differences between Tequendama deer and guinea pig for *δ*C_diet(en)_ and *δ*^18^O values, respectively (Table S2). Human individuals (*n* = 7) at Tequendama sampled for *δ*^13^C_diet(en)_ (range = −26.9 to −24.9*‰*; average = −26.3 ± 0.3*‰*) and *δ*^18^O (range = −6.6 to −3.9*‰*; average = −5.6 ± 0.2*‰*) were all taken from the burial concentration in zone T3 (ca. 8,830–6,090 cal BP). The average *δ*^18^O data of *H. sapiens* (5.6 ± 0.2*‰*) are close to both O. *virginianus* (−5.1 ± 0.2*‰*) and *Cavia* sp. (−6.2 ± 0.2*‰*).

Time-binned data from Tequendama enables exploration of faunal *δ*^13^C_diet(en)_ and *δ*^18^O data from sequenced zones of occupation T1–T4, representing a time span of 12,995–815 cal BP (Figures 3, S1, and S2). Other than displaying lower values in zone T3, *O. virginianus δ*^13^C_diet(en)_ and *δ*^18^O values remain fairly consistent across all temporal zones. Indeed, ANOVA testing of intra-site data from Tequendama *O. virginianus* show that there is no significant difference between *δ*^13^C_diet(en)_ (F(3,21) = 0.763, *p* = 0.53) nor *δ*^18^O (F(3,21) = 1.595, *p* = 0.22) values across zones of occupation (T1–T4). Averages of *δ*^13^C_diet(en)_ from *Cavia* sp. samples demonstrate consistent values when separated by zones of occupation and fall between −20.5 ± 0.2*‰* to −19.7*‰* ± 0.3, however their range in carbon isotope data broadens through time. Specifically, *Cavia* sp. samples exhibit a narrower *δ*^13^C_diet(en)_ range in the oldest zones of occupation T1–T2 (12,995–8,255 cal BP) when compared to zones T3–T4 (8,830–815 cal BP). *Cavia* sp. Observation of *δ*^18^O data also generally decrease through time where average values from oldest occupational zones T1 (−5.8 ± 0.2‰) and T2 (−5.3 ± 0.2‰) are higher than youngest zones T3 (−6.3 ± 0.2‰) and T4 (−7.1 ± 0.2‰). However, ANOVA testing of intra-site data from Tequendama *Cavia* sp. determine that these differences are not enough evidence to conclude that the *δ*^13^C_diet(en)_ (F(3,33) = 0.464, *p* = 0.71) or *δ*^18^O (F(3,33) = 1.843, *p* = 0.16) values across zones of occupation (T1–T4) are significantly different.

### Tequendama sequence: δ^13^C and δ^15^N from collagen

All *δ*^13^C_diet(col)_ values and C:N ratios from bone collagen extracted from Tequendama and Aguazuque samples are reported in Table 1. Only samples that had a C:N ratio between 2.9 and 3.6 and collagen yield >1% were plotted based on standard quality control measures.^62^ At Tequendama, only *O*. *virginianus* (*n* = 28) samples were analyzed for *δ*^13^C and *δ*^15^N due to sample availability. Across all zones, the average *δ*^13^C_diet(col)_ value is −23.1 ± 0.1‰ and the average *δ*^15^N_col_ value is 4.8 ± 0.1‰. The range in *δ*^13^C_diet(col)_ values from deer bone collagen for all occupational zones spans from −23.5‰ to −22.2 with values varying <1.4‰, except for the most recent zone T4 (>6,485 cal BP) that yielded a broader range of values from −26.5‰ to −22.6‰.

### Aguazuque sequence: δ^13^C and δ^18^O from enamel

At Aguazuque, *O. virginianus δ*^13^C_diet(en)_ values have a narrow range (−26.2 to −24.3*‰*) and an average of −25.5 ± 0.3*‰*. Deer *δ*^18^O values also produce a narrow range (−6.9 to −4.8*‰*) with an average of −5.7*‰* ± 0.2*‰* (Figure 3; Figure S3). Human individuals at Aguazuque (*n* = 4) have *δ*^13^C_diet(en)_ values from −25.6 to −16.9*‰* (average = −23.3 ± 0.3*‰*) and *δ*^18^O values from −6.4 to −4.3*‰* (average = −5.9 ± 0.2*‰*).

When compared temporally across the occupation zones, deer from zones A1-A2 (5,900–4,200 cal BP) have the lowest *δ*^13^C_diet(en)_ values. Amongst deer, the specimen from zone A2 has the highest *δ*^18^O enamel value. Human individuals at Aguazuque (*n* = 4) had *δ*^13^C_diet(en)_ values ranging from −25.6 to −16.9*‰* (average = −23.3 ± 0.3*‰*) and *δ*^18^O values ranging from −6.4 to −4.3*‰* (average = −5.9 ± 0.2*‰*). Broken down across occupational zones, the individual oxygen isotope value (−4.3*‰*) from the oldest zone A1 (5900–5650 cal BP) is higher than all other samples from overlying zones at the site and have a tight range of values from −6.4 to −6.3*‰*. Regarding human *δ*^13^C_diet(en)_ values, the majority of individuals sampled at Aguazuque have values lower than −25.0*‰*. However, one individual from Aguazuque zone A2 (4,400–4,200 cal BP) has a noticeably higher *δ*^13^C_diet(en)_ value of −16.9*‰*.

### Aguazuque sequence: δ^13^C and δ^15^N from collagen

At Aguazuque, deer *δ*^13^C_diet(col)_ (*n* = 8) have an overall average of −22.2 ± 0.1*‰* across all zones of occupation. Human postcranial elements (*n* = 11) were recorded from across all occupational zones and produced an inter-zone range of *δ*^13^C_diet(col)_ values from −24.1 to −15.4*‰* with an overall average of −23.2 ± 0.1*‰*. Although most *δ*^13^C_diet(col)_ values of humans at Aguazuque are similar, one individual from the youngest occupational zone A5 (2,900–2,750 cal BP) is higher, −15.4*‰*, than those from preceding zones.

### Site comparison

The stable isotope results (*δ*^13^C_diet(en)_, *δ*^18^O, *δ*^13^C_diet(col)_ and *δ*^15^N) from Aguazuque and Tequendama are compared in Figures 2, 3, and 4. Looking at all occupational zones, these composite data highlight that *δ*^13^C_diet(en)_ data for deer from Tequendama zone T4 (6,485–815 cal BP) and Aguazuque zone A5 (2,900–2,750 cal BP) temporally overlap and have the same average value of −25.8 ± 0.3*‰*. Meanwhile, the *δ*^13^C_diet(en)_ values of *O. virginianus* from older Tequendama temporalities are generally lower, with zones T2 and T3 having average values of −26.4 ± 0.2*‰* and −26.4 ± 0.3*‰*, respectively. Regarding deer *δ*^18^O, Aguazuque has a lower average value (−5.7 ± 0.2*‰*) than all Tequendama zones of occupation, excluding the oldest zone T1 value (−8.5 ± 0.3*‰*). Despite temporal variation of inter-site isotopic data among deer, ANOVA tests demonstrate that *O. virginianus δ*^13^C_diet(en)_ (F(4, 26) = 1.296, *p* = 0.30) and *δ*^18^O (F(4, 26) = 2.012, *p* = 0.12) values from Tequendama and Aguazuque are not statistically different between the occupational zones. When all isotopic values are compared independent of zones of occupations, there remains no significant difference in *δ*^13^C_diet(en)_ values (*t*-test; *t* = 1.8731, *p* = 0.09) of deer between the sites. However, results from *δ*^18^O values (*t*-test; *t* = −2.594, *p* = 0.01) suggest a significant difference in the values of deer between the sites.

Humans from Tequendama and Aguazuque (Figure 3) have similar overall site average *δ*^18^O values of −5.6 ± 0.2*‰* and −5.9 ± 0.2*‰*, respectively, and a *t*-test shows they are not significantly different (*t* = −0.50276, *p* = 0.64). However, more distinct values between the sites are seen in *δ*^13^C_diet(en)_. Excluding one data point, all human *δ*^13^C_diet(en)_ values from Tequendama are lower than those recorded at Aguazuque. At Tequendama, the majority of samples have *δ*^13^C_diet(en)_ values −26.1*‰* or lower which is reflected in the average of −26.3 ± 0.3*‰*. By contrast, samples from Aguazuque average −23.3 ± 0.3*‰* where all *δ*^13^C_diet(en)_ values are −25.6*‰* or higher. Most notably, the highest *δ*^13^C_diet(en)_ value, −16.9*‰*, originates from occupational zone A2 (4,400–4,200 cal BP) and is a considerably higher *δ*^13^C_diet(en)_ value than all other human components recorded at Tequendama. The median *δ*^13^C_diet(en)_ value for Tequendama is −26. 5*‰* (IQR = 0.5*‰*), and for Aguazuque it is −25.4*‰* (IQR = 2.4*‰*). A Mann-Whitney-U test (W = 29; *p* = 0.03) demonstrates a significant difference between the *δ*^13^C_diet(en)_ values of *Homo sapiens* at Tequendama and Aguazuque.

All *δ*^13^C and *δ*^15^*N* values from bone collagen are plotted in Figure 4 and divided by taxa, site and furthermore by occupational zones (12,995–815 cal BP; Figure S4). Deer samples at Tequendama have *δ*^13^C_diet(col)_ values ranging from −26.5 to −22.2*‰* with an average of −23.0 ± 0.1*‰*. These results are comparable to the *δ*^13^C_diet(col)_ values of Aguazuque deer that range from −21.5 to −22.9*‰* and average −22.2 ± 0.1*‰*.

## Discussion

### Refined site chronology

Advancements in the fidelity of direct dating techniques applied to archeological materials present an opportunity to refine the chronology of previously excavated sites and re-examine museum material. In the Sabana of Bogotá, recent publications focusing on minor re-evaluations of Aguazuque and Tequendama have contributed to the paleoenvironmental and temporal understanding of these sites.^63^^,^^64^^,^^65^^,^^66^^,^^67^ From Aguazuque, Carvajal et al.^63^ test the antiquity of the site utilizing electron spin resonance (ERS) spectroscopy and conclude that the mean age agrees with the original stratigraphic analysis put forth in Correal Urrego.^40^ Although this conclusion is based on a limited sample size, the alternative ESR dating methodology supports the original temporal binning of Aguazuque zones of occupancy, A4–A5. Moreover, a recent re-excavation of the site provides a single radiocarbon direct date^66^^,^^67^ from human burial contexts that generally aligns with the site chronology (Figure S2). Similarly, this previous re-excavation intended to provide a comprehensive stratigraphic and paleoenvironmental assessment of Tequendama.^64^ The authors of this earlier study present a radiocarbon date from a part of the stratigraphy defined as comparable to zone of occupation T2 and suggest a potentially broader temporal range for the Tequendama zone T2. Their interpretation of zone T2 is based on a single uncalibrated date and a reinterpretation of the site stratigraphy which highlights the need for increased dating efforts at Tequendama.

In this study, radiocarbon dates and Bayesian modeling help expand our understanding of human occupation at Tequendama as well as the Sabana of Bogotá region. Although results from these analyses (Figures S5 and S6) generally support the zones of occupation originally defined at Tequendama,^39^ our data provide some minor refinements in site chronology. For example, our results (samples OxA-43250 and OxA-43251) from the first zone of occupation align with the initially interpreted temporal range of zone T1 radiocarbon dates (13,410–10,690 cal BP).^39^ Results from Bayesian modeling present similar LP-EH temporal boundaries and suggest that zone T1 ranges from 12,995 to 10,645 cal BP. Notably, Correal Urrego and van der Hammen^39^ reported that there are no direct dates from the stratigraphy that comprise the zone of occupation T2, and infer the temporal range of T2 based on radiocarbon dates from under and overlying units as well as similar occupational features at the Sabana of Bogota site, El Abra. In a re-excavation of Tequendama, sampling of human remains interpreted to derive from the unit that comprises zone of occupation T2 yielded a radiocarbon date of 7,023–6,795 cal BP.^64^ This data is outside the previously defined temporal range of dates from zone T2 (10,690–8060 cal BP) and suggests a possible broader range for the zone of occupation. Albeit, the stratigraphic control and small radiocarbon sample size from this re-excavation introduce uncertainty and raise concerns about the comparability.

To better understand the fidelity of dating, we obtained multiple radiocarbon dates from zone T2. In this study, dates (samples OxA-43306; OxA-43307) support the original temporal description from the upper portion of zone T2.^39^ Results from Bayesian modeling present similar early Holocene temporal boundaries estimating that zone T2 ranges from 10,750 to 8,255 cal BP. From our study, a zone T3 radiocarbon date (sample OxA-43304) generally supports the original dates of zone T3 (8060–6490 cal BP) with the incorporation of a later end date. Specifically, results from Bayesian modeling present similar mid Holocene temporal boundaries, but estimate zone T3 ranges from 8,830 to 6,090 cal BP. Lastly, from the latest zone we present a date that likely represents the upper limit of zone T4 (sample OxA-43305) and possibly some intermixing of zones T3 and T4 at their boundary. Results from the Bayesian analysis estimate that zone T4 spanned from 6,485 until a boundary end date of 2,330 cal BP, or possibly until 815 cal BP. This range of mid to late Holocene dates is broader than that originally proposed by Correal Urrego and van der Hammen.^39^ However, those authors recognized the limitations of their sampling, and suggested that the units underlying and possibly comprising portions of zone T4 would represent a window of time from 6,000 to 2,500 BP. Due to limited temporal constraints, we acknowledge the likelihood of a wider occupational timeline for zone T4 and propose that it should include 6,485–815 cal BP until more precise dates can be obtained.

In our analysis, radiocarbon dates at Tequendama largely confirm the original chronology of the site and help revise the occupational history during the early to late Holocene. Moreover, the dates presented by our study, when compared to the original dates in a Bayesian model, provide confidence that the exhibiting radiocarbon chronologies for both sites are robust and broadly reliable. When compared to regional data, paleoenvironmental and isotopic patterns between Tequendama and Aguazuque help suggest differences in economic, settlement practices (see subsequent sections) and population dynamics. The latest suggested date of Tequendama aligns with reports of Herrera period ceramics onsite^39^ and the suggested range of early and late Herrera dates is supported by regional literature.^68^^,^^69^ Other research on radiocarbon chronologies in the Sabana of Bogota suggests that major population events took place during the mid-Holocene (ca. 6,000–3,800 ^14^C BP) and were influenced by paleoenvironmental changes that promoted dramatic regional depopulation and introduction of new populations.^70^ Results from our study do not contest these regional interpretations, however, they do provide additional chronological evidence of human activity at Tequendama during this time of demographic turnover. Still, the degree to which humans continuously or intermittently occupied the site still merits further investigation.

### Interpretation of human and fauna data

Stable isotope analysis of human and animal remains from Tequendama and Aguazuque provides valuable insights into dietary patterns and environmental conditions during the late Pleistocene and through the majority of the Holocene in Colombia. The fauna identified at Tequendama and Aguazuque are characteristic of LP-EH archaeological sites in the Sabana of Bogotá^71^ as well as modern C_3_-dominated subparamo and high-elevation Andean forest conditions.^43^ However, given the extensive nature of the modern savannas in the region, it is important to reconstruct the degree to which forest or grassland biomes were available to humans in the past and the extent to which early human occupants exploited plant and animal resources in the Sabana of Bogotá.

In Colombia, *δ*^13^C values of terrestrial C_3_ vegetation from the Andean region commonly range between −32‰ and −23‰,^36^ differing from the lower values of −37‰ to −24‰ reported from adjacent western Amazonian studies.^72^^,^^73^ *δ*^13^C_diet(en)_ data from *O. virginianus* (−28.8 to −24.3‰) at both sites align with expectations, representing a predominantly C_3_-browser diet with values indicative of environments from closed forest to more open woodland. This restricted *δ*^13^C range for *O. virginianus* suggests the consumption of locally available forest or grassland materials. Corresponding *δ*^13^C_diet(col)_ and *δ*^15^N values support these claims and the restricted range of these carbon values, as with C_3_ feeding animals, could suggest the preferential consumption of local C_3_ plant material (e.g., cold-adaptive Paramos grasses) with minimal incorporation of C_4_ grasses.^36^^,^^74^^,^^75^ The average *δ*^13^C_diet(col)_ of *O. virginianus* at Aguazuque is consistent (−22.2 ± 0.1*‰*) with that of Tequendama (−23.0 ± 0.1*‰*), and all isotopic values from deer specimens tell a similar dietary narrative of C_3_ plant material consumption in agreement with their isotopic niche. Some of the earliest evidence of hunting-gathering technology and subsistence specialization in the diet of Tequendama inhabitants comes from the namesake Tequendamiense lithics found in association with preserved cervid (e.g., *Mazama americana* and *Odocoileus virginianus*) remains.^39^^,^^76^ Similar taphonomic alteration and preferential utilization of *O. virginianus* has been documented at Tequendama^77^ and Aguazuque.^40^ In this study, *δ*^13^C_diet(en)_ data indicate heavy consumption of C_3_ components, plants and animals, incorporated into the diet of humans at Tequendama and aligns with this early use of deer in the Sabana of Bogotá.

As one of the most common taxa found at LP-EH archeological localities, *O. virginianus* are known to be opportunistic feeders and adaptable to varied landscapes as well as environmental conditions. This adaptability is crucial for *O. virginianus* to navigate dynamic and heterogeneous neotropical environments. Deer subsist almost entirely on C_3_ plants, but *δ*^13^C values obtained are dependent on the carbon isotopic composition of the deer’s diet and have a detailed record of dietary range^78^ and trophic shifts.^79^ Specifically, modern studies on *O. virginianus* commonly consider a *δ*^13^C value from collagen of −22.9*‰* as the upper endpoint for ungulates with a C_3_ only diet, whereas archaeological studies of white-tailed deer commonly range from −22.5 to −19.5*‰*.^10^^,^^80^^,^^81^ Exploitation of white-tailed deer intensified over the Holocene in Central^10^ and South America. However, displacement of white-tailed deer for guinea pig as a preferred prey species has been documented on the Sabana of Bogota at El Abra as early as mid-Holocene.^82^

Work from Martínez-Polanco^83^ is in agreement with this notion and reports that the inhabitants at Aguazuque likely focused on *Cavia* species rather than *O. virginianus* for meat consumption as well as a continued subsistence strategy dependent on plants. *Cavia* species are sensitive recorders of paleovegetation and act as a proxy for local vegetation due to their limited home ranges. In our study, Tequendama *Cavia* sp. represent a broader range of *δ*^13^C_diet(en)_ values in comparison to *O. virginianus*, attributable to differences in feeding ecologies, suggesting a more varied diet for this taxon and the probable incorporation of variable C_3_/C_4_ contributions from 8,830 until possibly 815 cal BP (Figure 3). This implies the existence of habitats favorable to C_4_ vegetation through the early to mid-Holocene and possibility of guinea pigs consuming wild-type plants supplemented in-part by human maintained C_4_ vegetation in the Sabana of Bogotá. Modern nutritional observations of *Cavia porcellus* in the Colombian Andes show predominant consumption of grasses (Poaceae). Furthermore, although guinea pigs do not directly compete against humans for maize,^84^ they have a high observed potential for supplement foraging^85^ and are an economic and dietary commodity. Incorporation of *Cavia* sp. into the LP-EH human diet is supported by biomolecular research. Application of aDNA research on *Cavia* species from archaeological sites Aguazuque and Checua clearly indicates the Sabana of Bogotá as a probable domestication hub producing genetically distinct taxa (ca. 6.0–2.0 ka) from modern populations, emphasizing the intense human-animal relationships in this region.^86^

Overall, when compared to data from Amazonia^87^ as well as South Asia,^42^ the animals at Tequendama and Aguazuque do not appear to indicate a significant influence of the canopy effect. The majority of deer samples consistent with open tropical forest or woodland at both sites, with some presence of tropical grasslands and clearings. Research from Sugiyama et al.^10^ argue that deer isotopic analyses can provide optimal proxies for documenting gradations in the range of human-animal encounters in the wild. *δ*^18^O values from Tequendama and Aguazuque would also support a mixed forest-woodland and grassland mosaic of moderate aridity. Higher *δ*^18^O values in the deer are consistent with its status as a non-obligate drinker.^45^ Human *δ*^13^C_diet(en)_ data from Tequendama and Aguazuque suggest that humans from periods 12,995 to 6,090 cal BP were primarily focused on the forest-woodland C_3_ end of the spectrum, consuming a mixture of both associated fauna and plants. The importance of these types of resources has been underscored by archaeobotanical studies around the Sabana of Bogotá and the tropical regions of Colombia (e.g., Palm).^9^ There is no evidence for humans exploiting closed canopy resources, however, and their values are more similar to those from humans living in the tropical forest mosaics of Panga ya Saidi (ca. 78.0 ka), than the rainforests of Sri Lanka^41^^,^^52^^,^^88^ though potential variability in regional *δ*^13^C baselines should be acknowledged.^33^^,^^36^^,^^89^

In our dataset, there is evidence for a potential expanded human dietary variability (*δ*^13^C_diet(en)_ = −16.9‰) indicated by an individual in Aguazuque zone A2 (4,400–4,200 cal BP), suggesting consumption of C_4_ resources likely due to increased incorporation of mixed-feeding guinea pigs or domesticated plant material into the dietary spectra. Our baseline sampling is limited, so it is possible both wild and domesticated plants played a major role in human diets.^38^ Contemporary isotope research demonstrates that the modern Andean ecosystem is C_3_ dominated with the majority of C_3_ vegetation ranging in *δ*^13^C values from −32‰ to −22‰, whereas the more restricted C_4_ taxa of Cyperaceae (i.e., *Bulbostylis junciformis*, *Blomia tropicalis*, *Cyperus brevifolia*, C. giganteus) and Poaceae (*Muhlenbergia cleefii*, *Paspalum bonplandianum*, and *Sporobolus lasiophyllus*) have values ranging from −16 to −11‰ and generally occur in the Andean lowlands.^36^^,^^90^ A dominance of C_3_ inputs may have led to dampened C_4_ values in the overall *δ*^13^C diet. Alternatively, higher *δ*^13^C values are best attributable to changing proportions of C_3_ and C_4_ plant consumption. From this study, the *δ*^13^C values suggest that C_4_ taxa are unlikely to have formed a considerable portion of deer, guinea pig or human diets suggesting that any increase in *δ*^13^C toward the C_4_ end of the spectrum is probable to be a result of maize incorporation. Comparable isotopic data from Aguazuque report two *δ*13C_col_ values (AZ 458-32 E5.3 = −10.46*‰*; AZ 458-32 E5.3 = −11.18*‰*)^91^ sourced from the youngest occupational zone A5 (2,900–2,750 cal BP).^40^ These values are higher than those from preceding zones, similar to the *δ*^13^C_raw_ value of individual A27-01 (−11.37*‰*) from this study, and suggest the integration of more C_4_ materials into human paleodiets at Aguazuque. Moreover, previous studies incorporating Bayesian stable isotope mixing models from LP-EH humans from the Sabana of Bogotá suggest this dietary change occurred ca. 3,000–2,800 cal BP when people consumed significantly more C_4_ plants and animal protein.^33^ In this study, our *δ*^13^C_diet(en)_ data in occupational zone A3 (4400–4200 cal BP) captures a strong C_4_ value from an Aguazuque human before this suggested dietary change and represents a possible earlier incorporation of C_4_ resources. Our data adds refined evidence of a transition to early agricultural practices suggested within the literature.^39^^,^^40^^,^^64^

The collective evidence provides a strong argument for the early increased incorporation of C_4_ plant material in the paleodiets of Aguazuque inhabitants. However, there remain alternatives that merit exploration. Importantly, previous studies at Aguazuque have recovered the partial shells of gastropods or bivalves as well as fish bone remains (e.g., *Grundulus bogotensis*)^40^^,^^65^ and possibly caiman (*Crocodylus acutus*). The Sabana of Bogotá region had a large Pleistocene lake that entirely dried up before the earliest occupancy of Tequendama, however intermittent periods of wetter climatic conditions partially restored portions of this lake system, including the nearby Lake Fúquene,^92^ whose freshwater resources would have likely been accessible. Consumption of freshwater fish, especially shallow-dwelling or carnivorous fish, could result in higher *δ*^13^C values^93^ like that of individuals A08-01 (zone A2) and A27-01 (zone A5) in our dataset, though more detailed ecological and baseline work would be needed to evaluate this. Additionally, the higher *δ*^13^Cdiet_(col)_ of A27-01 also has a slightly higher *δ*^15^N value. Although the dietary interpretation for Aguazuque humans with higher *δ*^15^*N* values is likely attributable to the consumption of terrestrial animals, it is possible portions of their protein resulted from the incorporation of fresh-water fish. Nevertheless, paleodiet studies caution that higher *δ*^15^N values may also result from the consumption of water-stressed terrestrial fauna.^94^ As an area with concentrated late Pleistocene through Holocene localities^70^ and evidence of intermittent occupations before large-scale regional sedentarism,^95^ the Sabana of Bogotá was a suitable landscape for cultural exchanges. Paleogenetic studies and demographic modeling carried out on human remains at both Tequendama and Aguazuque largely support genetic continuity, however, the authors suggest that more complex demographic processes like assimilation, admixture or sex-biased migrations hold potential.^96^ It is possible that some of the isotopic variation documented in Aguazuque human diets could be the result of intra-regional mixing of populations, cultural contributions or changes in subsistence practices.

Along with archaeobotanical examples of C_3_ plant materials found at Aguazuque like the tuber lbia (*Oxalis tuberosa*) and pumpkin seeds (*Cucurbita pepo*), Correal Urrego^40^ suggests the potential input of C_4_ domesticated plant material like maize (*Zea mays*) as a dietary constituent amongst site inhabitants. Although no archaeobotanical evidence for maize has been found at Aguazuque, there are remains of maize starch grains recovered from the calculus of humans recorded from cultural layers at regionally coeval sites scale like Chechua (5,720–5,580 cal BP).^95^ The increasing use of more varied plants and animals, or, perhaps more generally, habitats, from the early to mid-Holocene would be consistent with the reported presence of agroecologies like the sub-Andean forest site, San Isidro, in the Cauca Valley of Colombia^9^^,^^28^ and beginning of agriculture on the Sabana of Bogotá landscape during this period.^14^^,^^97^ Our data suggest that human adaptive strategies had strong emphasis on both plant and animal resources in the region. While the majority of cultivated plants in the Andean region belong to the C_3_ category, maize and amaranth, or kiwicha (*Amaranthus caudatus*), stand out as the only C_4_ plants cultivated regionally, exhibiting high *δ*^13^C values.^98^ Analyses of ancient plant remains, including phytoliths, have played a crucial role in elucidating the process of maize adoption in the neotropics.^99^ Some reported evidence of early maize consumption and cultivation in the Colombia Andean region dates back to at least 8.0 to 5.0 ka,^14^ where there is a clear consistent signal of C_4_ maize pollen suggesting crop production as well as promotion of food production and sedentism.

### Paleoenvironmental insights

Our observed increases in human dietary diversity may be linked to environmental changes in the region. Environmental change, driven in-part by fluctuations in atmospheric CO_2_ conditions^100^ and precipitation levels, drastically altered the plant composition of the northern Andes. Specifically, the majority of Andean C_4_ grasses (e.g., *Eragrostis*, *Muhlenbergia*, *Paspalum*, *Setaria*, *Schizachyrium* and *Sporobolus*) that originated in open savannas and lowland grasslands^90^ migrated upwards to the Andean highlands and paramos (2200–3500 m asl) during glacial conditions with low *p*CO_2_. However, these C_4_ grasses were out-competed by their C_3_ counterparts (e.g., species belonging to the genera *Aciachne*, *Agrostis*, *Calamagrostis*, *Chusquea*, *Cortaderia*, *Festuca*, *Poa*) and largely disappeared or were relegated to lower elevation and drier portions of the paramos and subparamos by the mid-Holocene.^101^ It is possible that the changes are also linked to increasing human forest clearance on the landscape with the expansion of sedentary settlements and food production by the mid Holocene. Inter-site comparison of deer *δ*^13^C values show they are not statistically different, whereas the differences in *δ*^18^O values are significant, demonstrating the consistency of this taxa as a herbivorous consumer and the ecological insights it provides into the spatiotemporal pervasiveness of wild C_3_ material in the Sabana of Bogotá. Additionally, this supports regional paleoenvironmental records reported from the late Pleistocene well into the Holocene. Higher *δ*^13^C values in deer from Tequendama and Aguazuque when compared to published modern faunal data,^78^ even following correction for the Suess effect (∼1.0‰^102^‰ to 1.5‰ in Andes,^20^ could be a result of habitat disturbance in conjunction with varied availability of Andean C_4_ grasses over the terminal Pleistocene to Holocene. What is clear is that results from this study provide evidence that humans utilized mosaic environments and had diets reflective of increased consumption of C_4_ materials, perhaps from the gradual dietary incorporation of maize during the mid to late Holocene.

Comprehensive reconstructions based on palynological^37^^,^^103^^,^^104^^,^^105^ and paleoecological^33^ evidence provide useful comparative data to explore environmental evolution of the LP-EH landscape in the Sabana de Bogotá region. Prominent vegetation change (e.g., increase in the mixed forest biome and mosaic environments), drier conditions, as well as variability in precipitation and temperature occurred around 8,300–5,800 cal BP. Tequendama taxa from earlier zones of occupation are coeval with this transition to drier conditions. However, more *δ*^18^O analyses may be needed to test for climatic changes. Around 5,800–3,800 cal BP, the region experienced another dry period of reduced precipitation and moderate temperature increase.^40^ At Tequendama and Aguazuque, climatic conditions and floral composition during the LP-EH are also explicitly studied through multi-proxy analysis and represented in the literature and suggest that the middle Holocene Hypsithermal period (8.0–4.5 ka) records a transition from humid to drier conditions.^40^ Higher *δ*^18^O *Cavia* sp. values from Tequendama zones T2–T3 could capture this transition to drier conditions. Meanwhile, the onset of the late Holocene records warmer and wetter environmental conditions,^37^ and this change is potentially reflected in the lower *δ*^18^O values in zone T4. Despite these fluctuations in *δ*^18^O data, there are no statistically significant differences in inter- and intra-site oxygen values from the studied taxa, however.

When focusing on particular taxa, statistical analyses of intra- and inter-site faunal isotope data from Tequendama and Aguazuque show no significant differences in *δ*^13^C and *δ*^18^O values which indicate relative environmental stability experienced by these exploited taxa across the terminal Pleistocene-Holocene boundary. Muted shifts in *δ*^18^O values across taxa in this study may indicate the incorporation of dietary sources across taxa is a more influential factor driving isotopic values. Changes in *Cavia* sp. diets, from a strictly C_3_ diet in the oldest Tequendama temporalities (Figure 3) to a more mixed diet with C_4_ contributions between the beginning of zone T3 (8,830 cal BP) and the end of zone T4 (2,330–815 cal BP) could be indicative of a mix of climatically-driven environmental changes toward more open habitats (e.g., paramos and montane forests,^105^ human intervention in local plant composition, and material changes in distribution of C_4_ plant material. Still, a general lack of statistically significant change in *δ*^13^C and *δ*^18^O values from sampled *O. virginianus* and *Cavia* sp. specimens suggest environmental stability across the spatiotemporal boundaries of the local mosaic environments that encompass Tequendama and Aguazuque in the face of changes in climate.

### Regional human occupation and paleoecology

The Sabana de Bogotá offers a unique window into human adaptations and paleoecology in northwestern South America. Although the region has a dynamic history of vegetation and climatic change, our 127 regional stable isotope (i.e., *δ*^13^C, *δ*^18^O, *δ*^15^N) data points from the Tequendama and Aguazuque localities demonstrate that the environment was relatively stable and sampled taxa experienced subtle changes in diet. The broadening of *δ*^13^C_diet(en)_ values of occupants from our sites suggests transition into more diverse diets and economies as a result of growing C_4_ contributions from maize or guinea pig consumption. The selective use of plant material during the LP-EH has been suggested to likely have preceded the development of horticulture as the main economic strategy in northwestern South America.^106^ By the late Holocene, a transition from more C_3_ to mixed C_3_/C_4_ diet and incorporation of deer and guinea pig is similar to that proposed by Delgado^33^ and likely a product of both cultural preference and resource accessibility overtime. Comparison of our data with previously published isotopic data facilitates a more comprehensive overview of these changes in line with regional context.

Nueva Esperanza, in the Sabana of Bogotá, provides perspectives from ca. 2,350–350 cal BP, partially overlapping with the youngest occupational zones features in this study. Bone collagen results from human remains of *δ*^13^C ($\bar{x}$ = −12.4‰, range = −19.1 to −9.2‰) and *δ*^15^N ($\bar{x}$ = 9.4‰, range = 8.0‰ to 11.7‰) in the youngest zones indicate a mixed C_3_/C_4_ diet with strong dependence on maize supplemented by meat consumption (e.g., deer and guinea pig) and C_3_ plants.^53^ Results of humans from Aguazuque (5,900–2,750 cal BP) in our study produced an average *δ*^13^C_diet(col)_ value of −23.0 ± 0.1‰ (range = −24.1 to −15.4‰). The observed *δ*^13^C_diet(col)_ values from our study are lower than those reported from Nueva Esperanza. This illustrates the pulsed nature of dietary transitions in the Sabana of Bogotá and the degree to which a mixed diet encompasses varied portions of C_3_/C_4_ components, plants and animals. Moreover, the range of *δ*^13^C_diet(en)_ values (−25.6*‰* to −16.9*‰*) from Aguazuque inhabitants support the incorporation of a wider range of C_3_ and C_4_ components. Regarding *δ*^15^N, *Homo sapiens* from Aguazuque range from 6.4‰ to 9.8‰ (average = 8.3 ± 0.1*‰*) with values generally increasing through time from zones A5 to A1. This wider range of *δ*^15^N values suggests more diverse dietary sources from preliminary reliance on plants to increased utilization of faunal protein.

Checua is another prominent early Holocene locality in the Sabana of Bogotá providing intermittent occupational evidence from ca. 9,500 until 5,000 cal BP, and insights into the onset of sedentism in the region.^107^ From an archeofauna standpoint, there is a large overlap in species composition from Checua and our sites of interest, including the utilization of *Cavia* and processing of *O. virginianus* postcranial bones. Furthermore, data from Checua reveal a rich history of plant processing from starch grain analysis of lithics^95^ highlighting the importance of maize exploitation in the Sabana of Bogotá. Archaeobotanical evidence from another early Holocene site in the region, Zipacon, is coeval with Aguazuque occupational zones A3–A4 and aligns with the potential management of plants including the calabash tree (*Crescentia cujete*), avocado (*Persea Americana*), sweet potato (*Ipomoea potatoes*) and maize (*Zea mays*) lending to the idea that early Holocene settlers were incorporating an array of C_3_ and C_4_ domesticated plants into their diets.^40^^,^^97^

More broadly, plant consumption, foraging economies,^20^ and animal management practices have been studied in the Peruvian Andes^13^ and provide important insight into domesticated landscapes. Research from the southern Andean Wilamaya Patjxa site features individuals preserved with ‘tool kits’ specialized for big-game hunting from the early Holocene (ca. 11.0–9.0 ka) and provides strong supportive isotopic evidence that these individuals had a mixed C_3_/C_4_ and animal diet just prior to the LP-EH transition.^108^ The LP-EH megafaunal extinction resulted in a massive ecosystem reorganization of fauna in the Colombian Andes^12^ and potentially promoted increased exploitation of surviving megafauna such as *O. virginianus*.

### Conclusions

Our study contributes paleoecological insights into the record of interplay between early human populations, flora, and fauna, as well as the changing LP-EH South America landscape. Systematic stable isotope analysis of humans at Tequendama and Aguazuque reveals a significant difference in *δ*^13^C_diet(en)_ values between the two sites and suggests heterogeneity of diet and incorporation of varied resources into diets over time. Specifically, isotopic values from Aguazuque capture the early incorporation of C_4_ resources, likely indicating the regional consumption of maize around 4,400–4,200 cal BP. In comparison, Tequendama inhabitants have more typical C_3_ values. However, further investigations are needed to explore the specific contributions of C_3_-feeding animals to this isotopic profile. Carbon and oxygen isotope data from analyzed taxa suggest environmental stability during the terminal Pleistocene to Holocene transition, though slight deviations hint at periods of varied aridity and support the presence of an overall local mosaic landscape with mixed open forest and savanna environment. Results from this study facilitate intraspecific examination of Tequendama and Aguazuque as well as a regional comparison across LP-EH archaeological sites and contribute to the refinement of isotopic changes through time within the Sabana of Bogotá. While our study enhances our understanding of dietary diversity and environmental stability in the neotropics from 12,995 to 815 cal BP, further research and expanded data collection are warranted to refine our interpretations and address the nuanced complexities of human-environment interactions in different parts of this region. These findings contribute to the broader discussion on isotopic variability, emphasizing the need for localized studies to capture the unique dynamics of specific ecosystems and their human inhabitants.

### Limitations of the study

Advances in chronological datasets,^109^^,^^110^ identification of human hunting strategies, and evidence of potential human-megafauna interaction^18^^,^^111^ are pushing back the earliest occupancy dates of humans in South America. It should be acknowledged that the majority of radiocarbon dates used in this study are sourced from 1970 to 80s research. While this alone does not necessarily diminish their reliability, it does raise concerns about comparability with dates obtained with AMS and advancements in pre-treatment protocols.^11^^,^^112^ Nonetheless, the sequence of published radiocarbon dates from Aguazuque^40^ and Tequendama^39^ remain widely referenced as the most comprehensive from these particular sites in the Sabana of Bogotá. Moreover, our comparison of dates acquired in this study with these original dates suggests that they provide a broadly reliable chronological reference for the occupational zones.

As our understanding of human antiquity in South America increases, so does the importance of researching the intricacies of human-environment and human-fauna relationships. Data from this study facilitate intraspecific examination of Tequendama and Aguazuque as well as a regional comparison across LP-EH archaeological sites and contribute to the refinement of isotopic ecologies within the Sabana of Bogotá. The selected taxa are well-represented in the region and offer insights into fauna associated with human occupancy from LP-EH, yet this is only part of the picture. Expanding isotope analysis across more taxa and archaeological localities is necessary to mitigate limitations due to sampling bias and paucity of comparative regional data. Therefore, more data are needed to determine the extent to which broadscale changes in climate are reflected in the local paleoecological record of studied sites.

We acknowledge the important δ^13^C_col_ and δ^15^N_col_ contributions from previous studies at Tequendama and Aguazuque.^65^^,^^91^^,^^113^ Nevertheless, the Sabana of Bogotá would benefit from more substantial local isotopic baseline studies incorporating diverse animal and plant material as well as water sources and bedrock geology in order to explore geographic provenance, migration and isotopic trends like those conducted in other tropical regions.^114^ Although this study provides isotopic evidence to reconstruct human and animal diets at a regional scale, extrapolation from other regions in northwest South America would be beneficial for a more holistic dietary history. Further integration of zooarchaeological, archaeobotanical, and paleoenvironmental records is also needed to study the onset of human influences on plants and animals and the degree to which these changes are human- or climatic-driven. Isotopic studies conducted on archaeological samples from museum collections and newly excavated sites can help reveal dynamic changes in human diet, mobility and culture within LP-EH early South American communities.

## Resource availability

### Lead contact

Further information and requests for resources should be directed to and will be fulfilled by the lead contact, Michael J. Ziegler (mziegler@gea.mpg.de)

### Materials availability

This study did not generate new unique reagents. The archaeological remains of Aguazuque and Tequendama are stored in the Instituto de Ciencias Naturales, Universidad Nacional de Colombia (ICN-UNC) collections. All stable isotope data generated in this study are available in the supplemental information section of this article or from the lead contact without restriction.

### Data and code availability

• All stable isotope and radiocarbon data generated in this study are available in the Main Text or supplemental information section of this article and listed in the key resources table.
• The article includes and analyses existing data from previous publications and listed in the key resources table.
• This paper does not report original code.
• Any additional information required to reanalzse the data reported in this paper is available from the lead contact without restriction.

## Acknowledgments

This research has been funded by the LASTJOURNEY project (ERC_Adv_ 834514), 10.13039/501100007601Horizon 2020, 10.13039/501100000781European Research Council. We thank the 10.13039/501100004189Max Planck Society for additional funding. All sampled specimens are registered with and approved by the Instituto Colombiano de Antropología y Historia (ICANH). Authorization of Archaeological Intervention Number #8371 issued on September 30, 2019 and Amendment Addendum No. 007 on March 22, 2022 corresponding to Entry Number #2022144200017392. We express our gratitude to German Alberto Peña and the ICN-UNC researchers for their academic insights and access to museum collections and lab facilities. We also want to extend our acknowledgments to Dovydas Jurkenas and Hans-Georg Sell at MPI-GEA for their collaboration and contributions to graphics and figures.

## Author contributions

Conceptualization, M.J.Z. and P.R.; Methodology, M.J.Z., P.R., C.C., and L.B.V.; Investigation, M.J.Z., J.I., and G.M.R.; Writing – Original Draft, M.J.Z. and P.R.; Writing – Review and Editing, M.J.Z., P.R., M.R., J.I., F.J.A., C.C., and L.B.V.; Supervision, P.R. and J.I.

## Declaration of interests

The authors declare no competing interests.

## STAR★Methods

### Key resources table

**Table:** 

REAGENT or RESOURCE	SOURCE	IDENTIFIER
**Deposited data**		

Stable isotope data of archaeological remains from Tequendama and Aguazuque, raw, analyzed and standards data	This paper	Table T1, Table S1 and Document S1
Radiocarbon chronology from Tequendama, raw and calibrated dates; dataset incorporates dates acquired from previous publications from both Tequendama and Aguazuque	This paper; Correal Urrego and van der Hammen^39^; Correal Urrego^40^	Table T2 and Table S3

**Software and algorithms**		

R	R Core Team https://www.r-project.org/	R Version 4.1.2; RRID: SCR_001905
R Studio	RStudio Team https://www.rstudio.com/	RStudio Version 2023.03.0 + 386; RRID: SCR_000432
IntCal20 Northern Hemisphere	Reimer et al.^61^	Calibration Dataset https://intcal.org/
OxCal	Bronk Ramsey^115^	OxCal Version 4.4 https://c14.arch.ox.ac.uk/oxcal/OxCal.html

### Experimental model and study participant details

This study reports on the stable isotope and radiocarbon analysis of archaeological remains. Non-human bone samples from Tequendama and Aguazuque used in this study were sampled from Instituto de Ciencias Naturales, Universidad Nacional de Colombia (ICN-UNC) collections. All archaeological samples were curated in Bogotá, Colombia, in sterile plastic bags and given unique specimen identifiers. Subsequent analysis of Holocene human skeletal remains sampled from Tequendama (*n* = 8) and Aguazuque (*n* = 15) were conducted at the Max Planck Institute of Geoanthropology in Jena, Germany. All necessary permits for this archaeological sampling and analysis were obtained from the Instituto Colombiano de Antropología y Historia (ICANH).

### Method details

#### Methods for sample selection and strategy

In this study, archaeological remains of *Homo sapiens*, *Odocoileus virginianus*, and *Cavia* sp. were sampled from vertebrate fossil collections curated at Instituto de Ciencias Naturales, Universidad Nacional de Colombia (ICN-UNC). A total of 127 samples from the Sabana of Bogotá sites, Tequendama (*n* = 98) and Aguazuque (*n* = 29), were isotopically analyzed in order to better understand inter- and intra-site variability amongst selected taxa (Table 1). Specifically, this robust dataset from Tequendama and Aguazuque consists of human (*n* = 23), cervid (*n* = 67), and cavia (*n* = 37) from occupational zones defined by historical excavations. The majority of teeth were sampled as either isolated specimens or, in some cases, as part of a mandible or cranium. When possible, both dental and postcranial elements were sampled for stable isotope analysis using enamel powder from teeth and preserved collagen from bone material. All faunal enamel and postcranial specimens analyzed from both sites were primarily identified to genus and species level based on previous publications focused on the Tequendama^39^^,^^116^ and Aguazuque^40^ excavations supported by ICN-UNC scientific staff using inhouse reference collections. Some remains are associated with previous studies that focused on cranial pathological conditions^117^ and morphological variation.^118^ Varied sample sizes amongst the targeted taxa are reflective of the composition and preservation of the zooarchaeological record in the different sedimentary layers. Selected taxa (*Cavia* sp. and *Odocoileus virginianus*) were focused on for analysis due to their pervasive occurrence, yet limited exploration of isotopic analysis, amongst Sabana of Bogotá fossil localities.

Amongst human specimens, second and third molars were preferentially sampled due to their standard formation process and mineralization at later stages of tooth development, in comparison to other teeth. This delayed development allows a degree of confidence that the isotopic value measured does not likely incorporate a pre-weaning signal resulting from breastfeeding, making these molars suitable for studying spatiotemporal variation in dietary and broader environmental patterns. Notably, human bone and enamel mineralisation occur at different times of life, with an average collagen turnover rate of approximately 1–4% per year for adult individuals (>20 years), leading to varied inter-tissue overlap.^119^ This approximate turnover rate depends on the measured bone element (i.e., rib vs. long bone) because some bones naturally form more rapidly than others. When considering multiple tissue samples from a single individual, isotopic comparison of enamel and bone likely represent an earlier and later period in the individual’s life, respectively, depending on the age of the individual at death. Regarding postcrania, the dense shaft portion of long bones (e.g., femur, tibia) were typically sampled due to their compact nature and overall increased degree of preservation. From Tequendama, a total of *n* = 8 human molars were sampled. Human remains were sampled from only zone T3 due to preservation and availability of documented burials. From Aguazuque, a total of *n* = 11 postcranial and *n* = 4 dental elements were sampled across all stratigraphically defined zones of occupation. All faunal and human remains were photographed before and after sampling for isotopic analysis. For access to these images please contact ICN-UNC collections and the lead author.

From neotropical caviomorph rodent specimens (i.e., *Cavia* sp.; *Cavia porcellus*), or guinea pigs, mandibular incisors were preferentially sampled from the collections. Feeding habit and tooth wear analysis demonstrate the physiological importance of hypsodont teeth in *Cavia* species,^120^^,^^121^ and the characteristic ever-growing nature of *Cavia* sp. incisors best capture a broad ecological history of the specimen when applying bulk isotope analysis. From Tequendama, a total of 37 *Cavia* sp. incisors were sampled across all stratigraphically defined zones of occupation. Specifically, zone T4 (*n* = 9) incisors, zone T3 (*n* = 14), zone T2 (*n* = 8), zone T1 (*n* = 6). Previous research endeavors from literature of Tequendama and Aguazuque faunal material were used as comparison in this study.

Standardised tooth development and replacement records of extant cervid taxa, *Odocoileus virginianus*, exist.^78^^,^^122^^,^^123^ Similar to other mammalia, *Odocoileus virginianus* replaces deciduous milk-teeth for permanent teeth which they will use throughout adulthood. Specifically, *O. virginianus* dentition dictates that first molars likely record a pre-weaning milk-dominated dietary signal, whereas fully erupted second and third molars better provide insight into sources of nutrition recorded during adulthood.^78^^,^^122^^,^^123^ Timing of tooth development further demonstrates that the morphologically distinct mandibular third premolar erupts concurrently with, or after, the third molar. Following this guide, both M_2_/M^2^ and M_3_/M^3^ were strategically, and PM_3_ were opportunistically sampled for bulk isotopic analysis. In regards to postcrania, dense and well-preserved bones were sampled to complement enamel samples.

#### Methods for stable carbon and oxygen isotope analysis from tooth enamel

Preparation of samples was conducted at the ICN-UNC laboratory facilities and prepared according to widely utilised bulk enamel collection methodologies.^124^ Human tooth enamel of the Tequendama specimens were prepared at the Globe Institute molecular biology labs in Copenhagen, Denmark. Specifically, tooth samples were surficially cleaned using gentle abrasion sand-blasters to remove any foreign material and expose a clean enamel sampling facet. Using a diamond-tipped drill, powder enamel was collected from the full portion of the tooth to capture an averaged bulk isotopic value. Moreover, the application of enrichment factors as an offset between *δ*^13^C values of faunal material and the consumed source material (i.e., vegetation) facilitates inter-taxonomic comparison of isotopic data and dietary habits. Nevertheless, it is important to accurately represent the potential enrichment factor when interpreting dietary patterns of fossil taxa due to considerable variation in interspecific *δ*^13^C enamel-diet enrichment.

Previous studies focused on early humans and fossil hominins tend to utilise an enamel-diet fractionation factor of 13‰–14‰.^54^^,^^55^^,^^56^^,^^57^ However, others have argued that these values may not be entirely appropriate for ubiquitously reconstructing diet, and suggest that a range of values closer to ∼10‰ could be more appropriate.^125^^,^^126^ In this study, we apply the most widely used enrichment factor (14.0‰) for our *Homo sapien* isotope data, due to its specificity as an enamel-diet factor for large-bodied mammals. We acknowledge there remains debate surrounding the impact that taxon-specific physical and physiological influences (body size and digestion processes; Tejada et al.^51^ have on enamel-diet enrichment values and recognise future research may provide refined values. We apply 14.5‰ as an enamel-diet factor for *Odocoileus virginianus* based on the corresponding recommended values for large-bodied ruminant fermenters in recent literature.^50^ Both rodent genera *Cavia* and *Rattus*, remain two of the most studied laboratory fauna in the world and research on modern domestics presents differences between dietary practices like intensity of caecotrophy^127^ and hindgut fermentation.^128^ There remains a lack of literature that exclusively investigates the enamel-diet factor of archeological *Cavia* sp. However, previous laboratory feeding studies investigating isotopic offset in the diet of rodents exist. These core studies report a *δ*^13^C enrichment of 9.5‰–9.7‰^58^ and 9.5‰–10.3‰.^59^ Subsequent palaeoenvironmental research takes this range of values, rationalising that an average fractionation value of 9.9‰ may be utilised in the reconstruction of paleodiet of ancient rodents.^60^ Further palaeodiet research on endemic rodents from Precolumbian Central America, including domestic *Cavia porcellus*, ascribes the same 9.9‰ enamel-diet enrichment value^49^ sourced from previous studies, and is what we assigned to *Cavia* sp. material in this study.

For human and cervid specimens, bulk sampling spanned from the basal-most enamel root junction portion to the occlusal surface. Although bulk isotopic analysis is less resolute than serial or micro-serial sampled specimens,^129^ the aim is to provide preliminary insights into dietary and climatic variability throughout the period of tooth formation. Amongst *Cavia* sp., the aradicular nature of their ever-growing incisors permitted only partial collection of powdered enamel samples via drilling and was supplemented with collection of chipped enamel fragments manually pulverised by mortar and pestle. On average, about 10–20 mg of powdered enamel was collected and stored in a 1.5mL Eppendorf Tube for transport to the Max Planck Institute of Geoanthropology laboratories in Jena, Germany. Here, powdered enamel samples were chemically prepped for isotopic analysis following in-house pretreatment protocol which utilises a 0.1M acetic acid bath. Fully prepped samples and standards were weighed out and analyzed on Gas Bench II Interface coupled to a Delta V Advantage Isotope Ratio Mass Spectrometer (IMRS). Results, relative to VPDB for carbon and oxygen, are recorded according to Szpak et al.^130^ and reported herein. Isotopic *δ*^13^C and *δ*^18^O values were compared against International Standards: IAEA-603 (*δ*^13^C = 2.5‰; *δ*^18^O = −2.4‰); IAEA-CO-8 (*δ*^13^C = −5.8‰; *δ*^18^O = −22.7‰); USGS44 (*δ*^13^C = −42.1‰); NBS18 (*δ*^13^C = −5.0‰; *δ*^18^O = −23.2‰) and In-House Standard MaxHorse (*δ*^13^C = −12.4‰; *δ*^18^O = −7.8‰).

#### Methods for stable carbon and nitrogen isotope analysis from bone collagen

Some bone samples were obtained as identifiable fragments (approximately 500–2000 mg), while others were sourced off a larger bone segment using a drill with a diamond tipped cutting wheel. Prior to collagen extraction, cleaning via sandblasting was carried out on the outermost portion of compact bones to remove any foreign material prone to surface contamination. The remaining bone samples were placed in 12 mL glass test tubes and underwent standard pretreatment with moderate modification according to in-house protocols. A process of bathing powdered and chipped bone samples in 10 mL of 0.5 HCL acid was repeated every 48 h until viable samples were obtained. After thoroughly rinsed with distilled water (MilliQ) water, 10 mL of pH3 HCL solution was added to the samples, covered and placed on a 70^॰^C heating block for 48 h. Once demineralized, the remaining solution was isolated using EEZE filters, frozen, and lyophilized. Percent collagen yield was calculated. Pristine collagen material and standards were weighed out and analyzed on an Organic Elemental Analyzer coupled to a Delta V Advantage IRMS. Results are reported in per mil (‰) and *δ*^13^C and *δ*^15^*N* values were calibrated relative to the VPDB and AIR. Specifically, *δ*^13^C and *δ*^15^*N* values were compared against International Standards: IAEA-C6/N2 (*δ*^13^C = −10.5‰; *δ*^15^N = 20.3‰); USGS40 (*δ*^13^C = −26.4‰, *δ*^15^N = −4.5‰) and in-house standard FISH (*δ*^13^C = −15.7‰; *δ*^15^N = −13.9‰). Each sample was run in duplicate with an analytical uncertainty of ±0.35‰ for *δ*^13^C and ±0.32‰ for *δ*^15^N calculated according to Szpak et al.^130^ (Table S1).

We then applied a discrimination factor to *δ*^13^C bone collagen values to better interpret dietary contributions. In this study, we considered the standard 3*‰ δ*^13^C discrimination value for strictly herbivorous fauna (*Odocoileus virginianus* and *Cavia* sp.) and 4*‰* for *Homo sapiens,* similar to other archaeological publications on food resource reconstruction (Fernandes et al., 2014), as well as those used in the interpretation of terrestrial resource dominated palaeodiets from global^131^ and Sabana of Bogotá regional contexts.^53^^,^^91^ Additionally, changes in *δ*^15^N from one trophic level to the next leads to increases in the *δ*^15^*N* values of collagen of consumers, with the dietary assumption that the higher *δ*^15^*N* values correspond to more consumption of animal protein. In this study, we assume a 3‰–5*‰*^52^^,^^132^ range of trophic shift values in our interpretations, as has been used in other global studies^133^ and regional literature specific to the Sabana of Bogotá.^53^

### Quantification and statistical analysis

#### Statistical analysis

All statistical analyses were performed in R version 4.1.2 with code provided (Data S1) and results summarised in the Supplemental Information, Table S2. In order to determine normality, Shapiro–Wilk tests were conducted. Normally distributed data were subjected to either an ANOVA or *t*-test, depending on the number of compared groups. Specifically, ANOVA tests were performed for normally distributed data comparing intra-site *δ*^13^C and *δ*^18^O data of *O. virginianus* and *Cavia* sp. across Tequendama zones of occupation. ANOVA tests were also performed for normally distributed data inter-site *δ*^13^C and *δ*^18^O data of *O. virginianus* and *Cavia* sp. across Tequendama with Aguazuque included as another zone of occupation. *T*-tests were performed for normally distributed data comparing inter-site *δ*^13^C and *δ*^18^O data of *O. virginianus* as well as *δ*^18^O data of *Homo sapiens* between Tequendama and Aguazuque. Similarly, *t*-tests were performed for intra-site comparison of *O. virginianus* and *Cavia* sp. at Tequendama. Whereas, corresponding *δ*^13^C data of *Homo sapiens* from Tequendama and Aguazuque that was not normally distributed underwent a Mann-Whitney-U test. Results from these analyses help determine any significant differences amongst taxa and zones of occupation and across sites.

#### Chronological analysis, radiocarbon dates and calibration

We accompany stable isotope data of Tequendama and Aguazuque, with refined chronologies based on radiocarbon dating. The previously defined chronologies of both sites are based on stratigraphic analysis and supported by ancillary radiocarbon dating acquired during initial excavations to define zones of occupation throughout the sites.^39^^,^^40^ In this study, we build upon these original temporal classifications as well as provide recently published and recently acquired radiocarbon dates to better understand any patterns in occupational history. Along with calibrating the dates provided by Correal Urrego and van der Hammen,^39^ we incorporate other available published dates (*n* = 2) sourced from a brief reexamination of both sites.^65^ Notably, in this study, we provide a more extensive dataset of radiocarbon dates (*n* = 6) that derive from faunal material (*Odocoileus virginianus*) within the ICN-UNC Tequendama collection and are directly comparable to the samples in the original publication (Table 2). These samples span across all occupational zones at Tequendama (T1–T4) and help examine the collective fidelity of all radiocarbon dates (Table S3) with updated Bayesian modeling approaches.

All radiocarbon dates from this study were obtained from the University of Oxford Radiocarbon Accelerator Unit, calibrated using the Northern Hemisphere IntCal20 curve according to Reimer et al.^61^ on the OxCal 4.4 platform^115^ and reported in cal BP to standardise published dates and facilitate temporal comparison across LP-EH sites. Similarly, all Bayesian analyses were conducted on the OxCal 4.4 platform and all model parameters, resulting models and summarised data are presented in the Methods S2. In total, two Bayesian models were run for Tequendama radiocarbon data. The first model included only dates originally provided by Correal Urrego and van der Hammen,^39^ and the second model added data from a previous publication^65^ and the direct dates provided from this study.

## Supplemental information

Document S1. Figures S1–S6 and Methods S1 and S2, and Data S1

Table S1. Measurement of standard uncertainty data run with stable isotope samples from Tequendama and Aguazuque, related to Table 1

Table S2. Summary of statistical analysis comparing stable isotope values (δ13C, δ15N and δ18O) and their results from Tequendama and Aguazuque, related to Figure 2, Figure 3 and Figure 4

Table S3. Calibrated radiocarbon dates and Bayesian modelling of archaeological remains at Tequendama from this study and those from re-excavations of Tequendama and Aguazuque where direct stratigraphic comparison is cautioned, related to Table 2
