# Supplementary material for: Human dietary diversity in the Colombian Andes at the terminal Pleistocene-late Holocene sites Tequendama and Aguazuque
Source: iScience. 2025 Jan 7;28(1):111624. doi: 10.1016/j.isci.2024.111624 (PMC11784782; doi:10.1016/j.isci.2024.111624)
Supplement: Document S1. Figures S1–S6 and Methods S1 and S2, and Data S1 [file mmc1.pdf]

iScience, Volume *28*

Supplemental information

Human dietary diversity in the Colombian Andes at the terminal Pleistocene-late Holocene sites Tequendama and Aguazuque

Michael J. Ziegler, Mark Robinson, Francisco Javier Aceituno, Gaspar Morcote-Ríos, Lorena Becerra-Valdivia, William C. Carleton, José Iriarte, and Patrick Roberts

METHODS S1: Background information expanding on the regional contexts of the Sabana of Bogotá in regards to its physiography, environmental and archaeological histories, especially as they apply to sites Tequendama and Aguazuque and an explanation of stable isotope analyses and proxies used in this study, related to Figure 1 and STAR Methods.

1.1 Physiography and Geology

Northwestern South America is not a homogeneous geographical region. Specifically, there are six natural physiographic regions that are commonly defined in Colombia, including: Andean, Caribbean, Pacific, Orinoquia, Amazonian and Insular (see Gómez Tapias et al.[134]). The Andean region corresponds to the prominent Andes mountain range that divides the Country into the Western, Central, and Eastern Cordilleras. The Sabana of Bogotá is an extensive high-altitude montane plain in the Eastern Cordilleras portion of the Andean physiographic region and is centrally located within the modern geographic borders of Colombia, department of Cundinamarca. Surrounded by the Eastern Cordilleras, the Sabana of Bogotá is situated at an average altitude of 2,650 m asl.[135] As part of the heterogeneous Andean region, the Sabana of Bogotá region is composed of multiple terrestrial biomes including savanna grasslands, páramo and various forestscapes (i.e., moist, montane, dry; **Figure 1**). Classification of these biomes are detailed in high-resolution modern ecoregion maps (e.g., Olson et al.[136]) that can be used in comparison to proposed biomes based on plant composition during the Last Glacial Maximum.[37,137]

Geologically, a semi-continuous and important record of sedimentary deposits exist in the Eastern Cordillera in the northern Andean region and have been detailed from through Quaternary.[138] The Sabana of Bogotá mainly comprises the Plio-Pleistocene Tilatá and Sabana formations within the Magdalena basin. The majority of archaeological sites that overlie these formations consist of reworked lacustrine deposits from the Humboldt palaeolake and relic Pleistocene–Holocene alluvial sediments. Within the Sabana of Bogotá, both Tequendama and Aguazuque are situated at similar altitudes at 2,570 m asl and 2,550 m asl, respectively. Despite geographic proximity and similar high-altitude positioning, Tequendama is classified as a dry montane forest, whereas areas at similar elevation to Aguazuque have been described as predominantly wet rainforest.[59]

1.2 Environmental History

The neotropical regions of the Americas are the most species-rich area on Earth and this longstanding history of biodiversity is a result of prolonged biotic interchange and environmental change.[139] Northwestern South America has been the focus of palaeoenvironmental studies, championed largely in part by the initial work of Thomas van der Hammen[35,111], and supplemented by subsequent palynological[37,93,95], geomorphological[141] and plant isotope[36] analyses in order to contribute palaeoclimatic and palaeontological data covering the LP-EH. Importantly, a large portion of these studies centre around the Sabana of Bogotá region of interest as well as the peripheral Andean Cordillera areas. Reviews of the palaeoenvironmental history of Sabana of Bogotá and northwestern South America during the Quaternary are available.[82,142] During the LP-EH, the Sabana of Bogotá region experienced repeated environmental changes with varying degrees of intensity in line with changes in temperature and aridity. These climate-dependent fluctuations could have led to substantial ecological modifications, impacting both plant and animal communities.

During the Last Glacial Maximum ca. 20.5–19.5 ka through the Early Lateglacial 17.5–16.5 ka interval, vegetation cover in the region was more indicative of cold and dry conditions, and notably in high-altitude areas (>2500m), this shift resulted in an increase of the cool grassland shrub Páramo biome.[29] The Guantiva Interstadial (15,000–13,000 cal BP) meanwhile is associated with an increase in average annual temperature and precipitation levels as well as expansion of forest over the páramo denoted by the presence of Andean forest genera *Alnus, Quercus, Myrica,* and *Myrsine*.[93,94,143] Altitudinal movement of the upper forest line (~400–500m lower than Guantiva Interstadial levels) continued during the onset of cooler and dryer conditions during the El Abra Stadial (13,000–10,800 cal BP) and the Sabana of Bogotá saw a replacement of forests with subpáramo vegetation (e.g., family Fagaceae)[35,37] and more open páramo grasslands (e.g., Poaceae and *Tubuliflora* spp. (Asteraceae).[93] Biome reconstructions by Marchant et al.[37], indicate that there was marked expansion of mesic vegetation around 10.5 ka at higher elevation where cool grasslands contracted and mixed forests predominated. During the early Holocene (8,300–5,800 cal BP)[33], climate shifted again and the region experienced a sharp increase in average temperature and dry environmental conditions evident by flora composition (e.g., Cactaceae; *Sapium* (Euphorbiceae), leading to the corresponding lowering of water table and drying of lakes and swampy areas[93] and expansion of mixed forest environments.[37] Following this, Delgado[33] reports that the region experienced a particularly dry period, reduced precipitation and general increase in temperature just prior to the onset of climatic conditions similar to today, beginning *ca*. 2,500 cal BP.

1.3 Archaeology and Site History

The past decade has witnessed a proliferation of studies on possible initial settling pathways of humans in Colombia[29,32] due to its geographical positioning as the first territory likely to have been encountered on the South American continent. Nevertheless, comprehensive reviews of neotropical savannas in northwestern South America demonstrate that these regions during the LP-EH are understudied and merit further investigation.[2] Within the Sabana of Bogotá, archaeological sites like Tequendama and Aguazuque are great candidates for systematic isotopic analysis due to their status as sustained LP-EH settlements, and therefore provide an opportunity to further elucidate the palaeoecological history of the region. Given the extensive nature of the modern savannas on the Sabana of Bogotá, it is important to reconstruct the degree to which forest or grassland biomes were available to humans in the region in the past and the extent to which early human occupants exploited plant and animal resources in the Sabana of Bogotá. Seminal work carried out in the 1970-90s detailed LP-EH human occupation at Tequendama based on the presence of lithic artefacts, human and faunal bone remains and plant material. Meanwhile, initial reports from Aguazuque recorded a large number of human burials, faunal remains and carbonised plant material.

Tequendama (4°31′59.881″ N; 74°16′30.895″ W and 2570 m asl)[39,59] is an archeological rock shelter located at and has been excavated since the late 1970s and historically interpreted as a LP-EH site intermittently used by early hunter-gatherer societies who were mobile and potentially had a diet that incorporated high levels of animal protein[39]. The site has fractured white-tailed deer bones that demonstrate burning, cut-marks and evidence of the processing of faunal material. An initial report by Correal Urrego and Van der Hammen[39]) provides radiocarbon dates of 13,525 cal BP from the earliest contexts associated with human activity (e.g., lithics fragments) and continues well into the Holocene. This history of inhabitants is supplemented with occupational zones (T1–T4) that are defined

by geological contexts and which vary in age, concentrations of human burials, faunal specimens and ceramic artefacts.

Aguazuque (4°36 ′32.200″ N y 74°16′50.375″ W and 2550 m asl)[40,59] is an open-air archeological site located ~8.5 km north from Tequendama and dated from 5,900–2,800 cal BP[40] with occupational zones (A1–A6). Contrary to Tequendama, Aguazuque is interpreted as having a potentially greater dependence on plant material and a more sedentary lifestyle accompanied by a higher social complexity.[40] Subsequent publications focus on morphological analysis of bones from burials and offer insights into ortho-dental information of Aguazuque human populations.[106] A taxon-specific report illustrates the relevance and legacy of white-tailed deer hunting at the site and suggests that other taxa were important contributions to the diet of humans, possibly including guinea pig, *Cavia* sp.[73] Moreover, Martínez-Polanco[144] suggested that *Cavia* sp. remains at Aguazuque could provide indirect evidence of sustained interaction between humans and guinea pigs and be an integral region demonstrating South America as a domestication centre.[76]

Between the two sites, there are some cultural similarities in terms of fauna present, bone tools, and ceramics. In addition to *Odocoileus virginianus* and *Cavia* sp., both sites show the presence of the remains of taxa such as: Brocket deer (*Mazama americana)*, Nine-banded armadillo (*Dasypus novemcinctus*), Paca/Agouti (*Agouti taczanowskii*), Coatis (*Nasuella olivacea; Nasua nasua*); Lesser anteater (*Tamandua tetradactyla*); and possibly Puma (*Felis concolor*). The majority of bone tools (i.e., perforated knives, scrapers daggers) are morphologically similar across the sites and composed largely *Odocoileus virginianus* bones. The ceramics from both Tequendama and Aguazuque are also classified as Herrera age ceramics.

O. virginianus and *Cavia* sp. species are the most common from both sites, but their estimated concentrations per zone of occupation vary (see source literature for more details on fauna at Tequendama[39] and Aguazuque.[40] For example, the following concentrations are based on samples from 3 different grids to give an idea of the general faunal composition at Tequendama: zone T1: 32% Deer; 6% Cavia, zone T2: 5% Deer; 33% Cavia, zone T3: 17% Deer; 57% Cavia and zone T4: Deer and Cavia sp. are the only taxa recorded from these test grids, but they do not provide exact estimated percentages. At Aguazuque, the frequency of *O. virginianus* and *Cavia* sp. is reported by zone of occupation as percentages compared to other excavated vertebrate taxa: zone A5 (Strata 5.2): 86% Deer; 13% Cavia, zone A4 (Strata 5.1): 79% Deer; 20% Cavia, zone A3 (Strata 4.2): 80% Deer; 18% Cavia, zone A2 (Strata 4.1): 76% Deer; 22% Cavia and zone A1 (Strata 3): 81% Deer; 17% Cavia.

Although fauna represented at Tequendama and Aguazuque are diverse, but *O. virginianus* and *Cavia* sp. have the highest taxonomic concentration through all zones of occupation. We acknowledge that these two taxa do not represent the entire spectra of local animals present during periods of human occupation, however, their spatiotemporal pervasiveness permit comparative sampling throughout all zones of occupation. Depending on availability, future studies would benefit from the analysis of a wider variety of fauna to further investigate other palaeoenvironmental perspectives and trophic interactions as well as contribute to a more refined regional isotopic baseline.

1.4 Stable Isotope Background

Stable isotope analysis is a well-established analytical technique in neotropical archaeology that is widely recognized for its utility in studies assessing palaeodiet[13,27,44,45,117],

palaeoclimate[4,146,147], and palaeoenvironment across a variety of spatiotemporal contexts. Amongst more forested C_3 environments, isotopic differentiation can be partially attributed to the 'canopy effect,' which influences $\delta^{13}C$ values of plants relative to their positioning within the vertical profile as well as variation of forest density; having the lowest values occur nearest to the ground.[46] Furthermore, recent advances in isotope ecology also demonstrate that $\delta^{13}C$ enrichment varies taxonomically amongst mammalian fauna based on body mass and digestive physiology.[51] Valid hypothetical models delimiting the percentage of vegetation that utilises C_3 vs C_4 pathway across the South American continent have proposed strong separation[43,79] amongst physiographic regions. Analogously, this partitioning of carbon-isotopic values is further supported by the work of Mora and Pratt[36] who highlight the altitudinal distribution of most archetypal plant species in and around the Eastern Cordillera of Colombia. In Colombia, $\delta^{13}C$ values of modern terrestrial C_3 vegetation from the Andean region commonly range between -32‰ and -23‰[36], differing from the lower values of -37‰ to -24‰ reported from adjacent western Amazonia studies.[62,63] These proposed $\delta^{13}C$ ranges are based on representative South American vegetation and their compositional differences in isotopic values can be recognized as distinct inter-environmental boundaries.

Oxygen isotope data ($\delta^{18}O$) are often utilised to explore relationships between trends in precipitation, aridity, seasonality and climate.[48] In general, oxygen isotope values of terrestrial mammal enamel (hydroxyapatite) derive from water consumed during life, with a relatively smaller contribution originating directly from food and respiration of atmospheric oxygen.[148,149] Non-obligate drinking herbivores, including representative deer taxa in this study, primarily acquire their water from vegetation. Therefore, it is thought that *Odocoileus virginianus* $\delta^{18}O$ is predominantly influenced by the $\delta^{18}O$ composition of the leaves consumed as a water source, making it particularly sensitive to climatic changes which might influence plant evapo-transpiration.[45] In the Colombian Andes, spatial and temporal distribution of $\delta^{18}O$ isotopic values have been attributed to variability in water sources including atmospheric input from surrounding Atlantic and Pacific oceans, evapotranspiration from the Amazonian basin, orographic filtering of winds and moisture recycling in the Andes Mountains, and Intertropical Convergence Zone seasonality.[150] Notably, there is an inverse relationship related to tropical rains and altitude[151] where comparatively low $\delta^{18}O$ values can be observed in high-altitude precipitation. Today, surface water $\delta^{18}O$ in the vicinity of Bogotá is heavily influenced by the isotopic value of rain and ranges from -5.2 to -11.5‰.[152]

Along with enamel, postcranial elements were sampled from selected fauna and human individuals in order to analyse $\delta^{13}C$ and $\delta^{15}N$ isotopes from bone collagen. Stable carbon isotope analysis of bone collagen permits exploration into relative reliance of dietary components. Specifically, $\delta^{13}C$ and $\delta^{15}N$ values may be a decent predictive tool for estimating the percentage of animal protein consumption as well as C_3 and C_4 resource contributions from plants in the diet of human agriculturalists.[54,153] Sensitivity of $\delta^{13}C$ and $\delta^{15}N$ values to variation in precipitation and diagenetic alteration are noted. This is especially true in tropical regions where preservation is limited by soil acidity and hydrological activity[42] and well documented in postmortem alterations of human burials.[154] Even though interpretation of archeological remains can be limited by diagenesis, the application of stable $\delta^{13}C$ and $\delta^{15}N$ isotopes from bone is an essential aspect of archaeological research and an indispensable tool for unravelling the history of humans and animals in LP-EH South America. When applied successfully, analysis of nitrogen stable isotopes provides additional resolution in the reconstruction of palaeodiet by providing insights into the trophic level position of human individuals, where a baseline can be established.[88,155] Early isotopic literature reports an approximate average $\delta^{13}C$ discrimination factor value of 0–2‰ and up to

2–5‰[127,156] relative to their diet (see review[157]). However, more recent reports caution for differences among taxonomic classes.[158] In this study, we considered the standard 3–5‰ $\delta^{13}C$ discrimination value and apply a 3‰ value for strictly herbivorous fauna (*Odocoileus virginianus* and *Cavia* sp.) and 4‰ for *Homo sapiens* similar to other archaeological publications on food resource reconstruction[159] as well as terrestrial dominated palaeodiets from global[131] and Sabana of Bogotá regional contexts.[53] Additionally, accumulation of $\delta^{15}N$ from one trophic level to the next leads to enrichment of the collagen in its consumer, with the dietary assumption that the higher $\delta^{15}N$ values correspond to more consumption of animal protein. Most literature reports an $\delta^{15}N$ enrichment value ranging from approximately 3–5‰[52,132] or even 6‰ from controlled dietary studies in humans.[160] In this study, we consider this range of values in our interpretations and assume an average enrichment factor within this 3–5‰.

Previous Sabana of Bogotá literature focused on the development of agriculture and analysis of stable isotope measurements from human remains[33] provide important information about the dietary diversity[11] and plant resources[81] used by these early human populations in the region. Coupled with palaeopedological analysis and regional palynological records, results from Vega et al.[57] suggest that there is a possible shift from hunting and gathering strategies towards more plant use at Tequendama during the early to mid Holocene. While the authors report that sampled fauna all have similar diets, they suggest an individual at Tequendama demonstrates a potential shift from C_3 to a more mixed diet. Nevertheless, this existing work is based entirely on 9 samples from human, deer, and guinea pig with a clear need for isotopic study across taxa and strata to establish a more refined spatiotemporal record of changes in environmental conditions and diet from LP-EH sites on a broad temporal scale. Results from this study utilise stable isotope analysis of Tequendama and Aguazuque to determine significant changes in $\delta^{13}C$, $\delta^{18}O$ and $\delta^{15}N$ amongst archaeological remains by zones of occupation. All estimated dates within the following figures are based of Bayesian modelling conducted as part of this study. The zones of occupation have been modeled as uniform phases using OxCal4.4[113, 114], reported in cal BP utlier analysis and checked for outliers.[161]

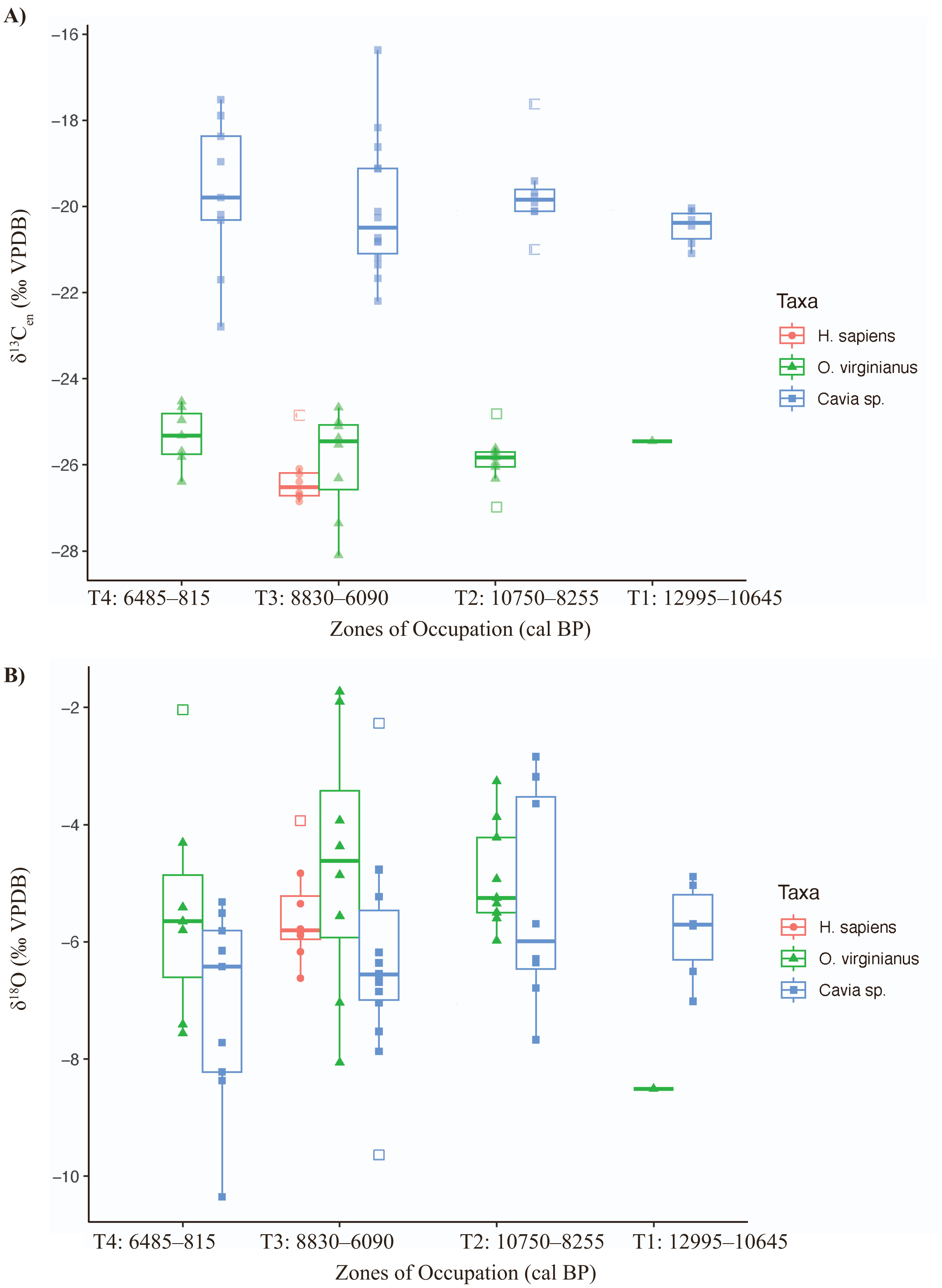

FIGURE S1: Boxplot of stable isotope data from the enamel of human and selected archaeological taxa at Tequendama by zone of occupation, related to Figure 2 and Figure 3. (**A**) $\delta^{13}C_{diet(en)}$ data from human, deer and guinea pig help illustrate differences in carbon isotope values and better interpret the consumer's diet. (**B**) Corresponding $\delta^{18}O$ stable isotope data from sampled taxa help illustrate differences in oxygen isotope values and better interpret water consumption or patterns in environmental conditions.

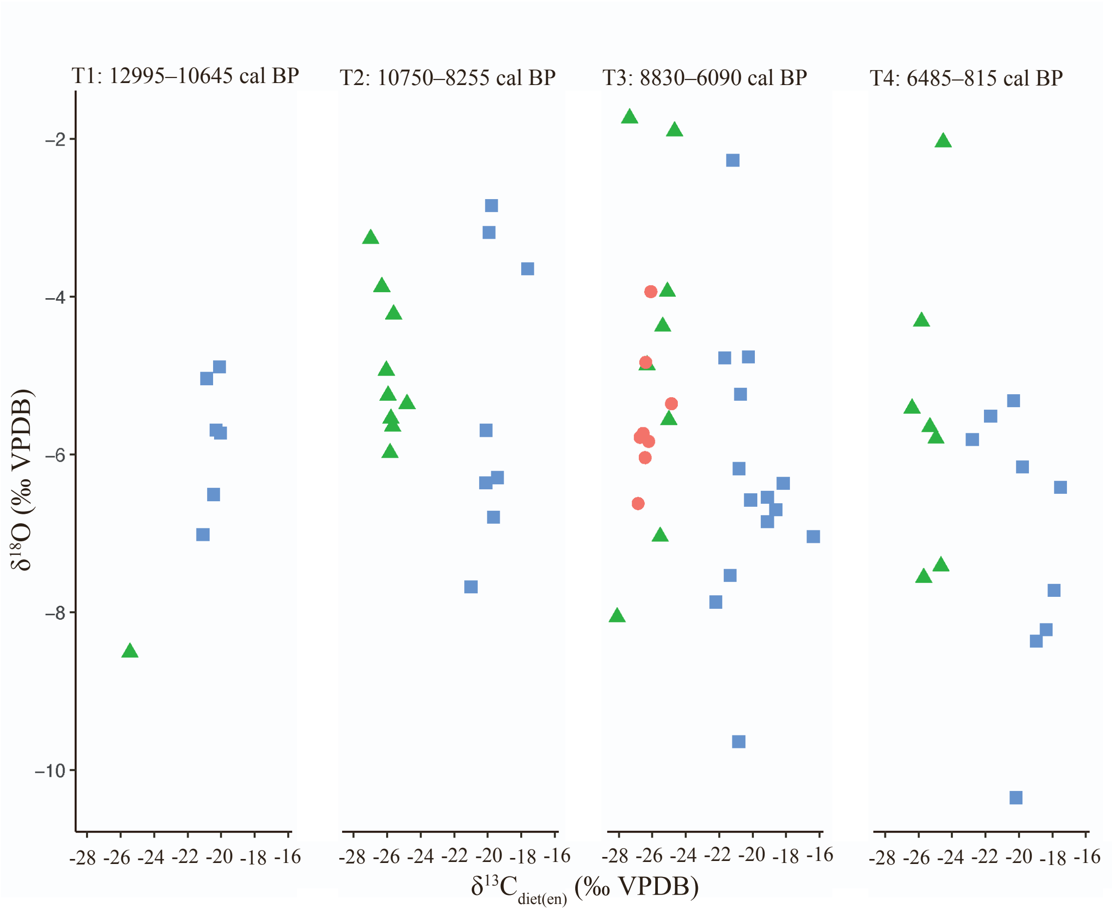

FIGURE S2: Bivariate Scatterplot of stable isotopes from the enamel of selected archaeological taxa at Tequendama by zone of occupation, related to Figure 2 and Figure 3. δ^{13}C isotopic data has been corrected for fractionation and plotted as $\delta C_{diet(en)}$ to investigate potential dietary patterns and plotted against δ^{18}O values in a successive time-series.

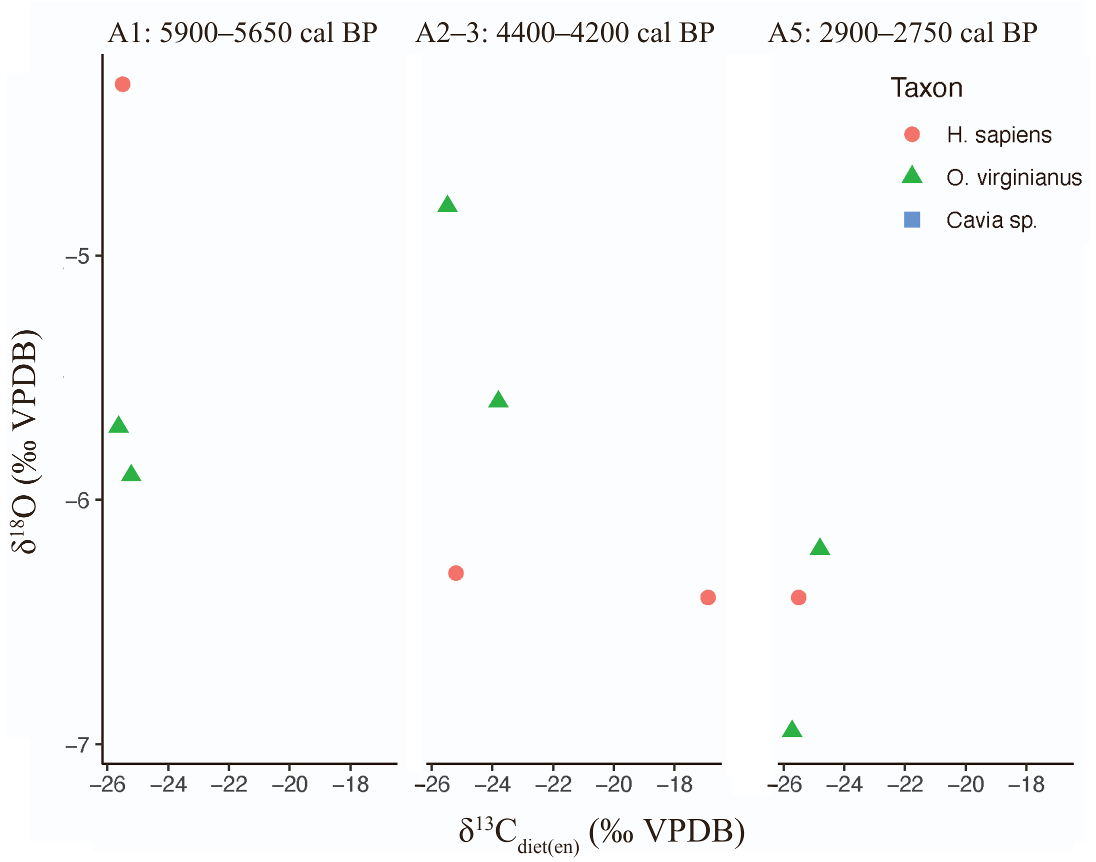

FIGURE S3: Bivariate Scatterplot of stable isotopes from the enamel of selected archaeological taxa at Aguazuque by zone of occupation, related to Figure 2 and Figure 3. $\delta^{13}C$ isotopic data has been corrected for fractionation and plotted as $\delta C_{diet(en)}$ to investigate potential dietary patterns and plotted against $\delta^{18}O$ values in a successive time-series.

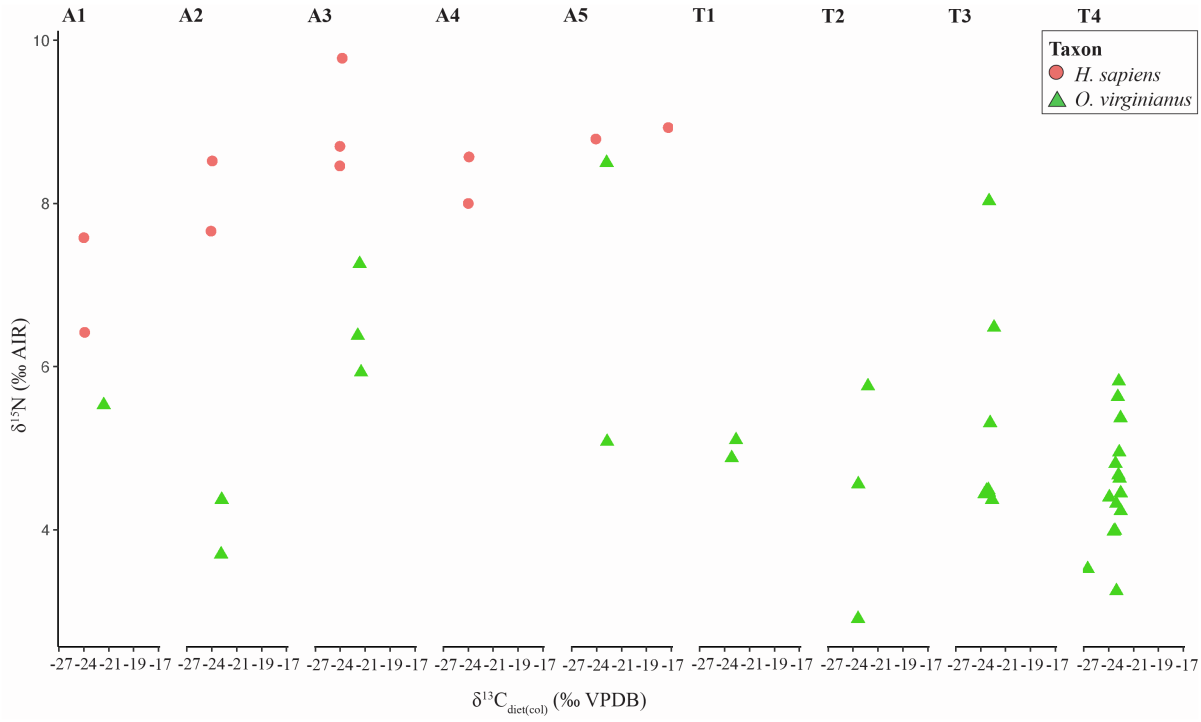

FIGURE S4: Bivariate Scatterplot of stable isotopes from bone collagen of selected archaeological taxa at Tequendama and Aguazuque by zone of occupation, related to Figure 4. $\delta^{13}C$ isotopic data has been corrected for fractionation and plotted as $\delta C_{diet(en)}$ and plotted against $\delta^{15}N$ values in a successive time-series where A1 (5900–5650 cal BP), A2-A3 (4400–4200 cal BP), A4 (3750–3550 cal BP), A5 (2900–2750 cal BP); T1 (12995–10645 cal BP), T2 (10750–8255 cal BP), T3 (8830–6090 cal BP) and T4 (6485–815 cal BP).

DATA S1: R script code associated with statistical tests of stable isotope data from Tequendama and Aguazuque, related to Table 1, Table S2 and STAR Methods.

#Tequendama Deer Across Zones T1-T4: Carbon from Enamel
```
shapiro.test(TeqDeerStats$dC13)
LM1 <- lm(dC13 ~Zone, TeqDeerStats)
aov(LM1)
summary(aov(LM1))
```

#Tequendama Deer Across Zones T1-T4: Oxygen from Enamel
```
shapiro.test(TeqDeerStats$dO18)
LM2 <- lm(dO18 ~Zone, TeqDeerStats)
aov(LM2)
summary(aov(LM2))
```

#Tequendama Cavia Across Zones T1-T4: Carbon from Enamel
```
shapiro.test(TeqCaviaStats$dC13)
LM3 <- lm(dC13 ~Zone, TeqCaviaStats)
aov(LM3)
summary(aov(LM3))
```

#Tequendama Cavia Across Zones T1-T4: Oxygen from Enamel
```
shapiro.test(TeqCaviaStats$dO18)
LM4 <- lm(dC13 ~Zone, TeqCaviaStats)
aov(LM4)
summary(aov(LM4))
```

Tequendama Deer vs. Cavia: Carbon from Enamel
```
shapiro.test(TeqDeerCaviaStatsReview2$dC13)
wilcox.test(dC13 ~Taxa, TeqDeerCaviaStatsReview2)
```

Tequendama Deer vs. Cavia: Oxygen from Enamel
```
shapiro.test(TeqDeerCaviaStatsReview2$dC13)
t.test(dO18 ~Taxa, TeqDeerCaviaStatsReview2)
```

Tequendama vs. Aguazuque Deer Across Zones T1-T4 with AGZ Zone: Carbon from Enamel
```
shapiro.test(TeqAqzDeerStats$dC13)
LM5 <- lm(dC13 ~Zone, TeqAqzDeerStats)
aov(LM5)
summary(aov(LM5))
```

Tequendama vs. Aguazuque Deer Across Zones T1-T4 with AGZ Zone: Oxygen from Enamel
```
shapiro.test(TeqAqzDeerStats$dO18)
LM6 <- lm(dO18 ~Zone, TeqAqzDeerStats)
aov(LM6)
summary(aov(LM6))
```

Tequendama vs. Aguazuque Deer: Carbon from Enamel

shapiro.test(TeqAqzDeerStatsReview1$dC13)
t.test(dC13 ~Site, TeqAqzDeerStatsReview1)

Tequendama vs. Aguazuque Deer: Oxygen from Enamel
shapiro.test(TeqAqzDeerStatsReview1$dO18)
t.test(dO18 ~Site, TeqAqzDeerStatsReview1

Tequendama vs. Aguazuque Human: Carbon from Enamel
shapiro.test(TeqAqzHumanStats$dC13)
wilcox.test(dC13 ~Zone, TeqAqzHumanStats)

Tequendama vs. Aguazuque Human: Oxygen from Enamel
shapiro.test(TeqAqzHumanStats$dO18)
t.test(dO18 ~Zone, TeqAqzHumanStats)

METHODS S2: Bayesian model of the radiocarbon dates available for the archeological site Tequendama and those provided by this study help refine the chronological history of human occupancy at the site and contributes to regional settlement patterns, related to Table 2, Table S3 and STAR Methods.

1.1 Notes on OxCal Code associated Bayesian Model, S5

In this model of Tequendama, only dates from Correal Urrego and van der Hammen[39] are used in order to: 1) calibrate the originally published radiocarbon dates, 2) test the original temporal boundaries through modeling of the zones of occupation, 3) update the occupational history of the site and 4) provide a standard chronological framework to compare with newly dated material. In the `Outlier Model`, default options have been used for all samples. Results indicate there are no outliers in this sequence. Model outputs;
-Zone T1: Place the start of the `Phase 1` from 13493–12536 and end around 11560–10379 cal BP with the `R_Date Function` estimating an age range of 13085–10876 cal BP and `Interval Function` duration of 1192–2855 years.
-Zone T2: Place the start of the `Phase 2` from 11560–10379 and end around 9650–7970 cal BP with the `R_Date Function` estimating an age range of 11137–8297 cal BP and `Interval Function` duration of 1195–3411 years.
-Zone T3: Place the start of the `Phase 3` from 9650–7970 and end around 6772–4353 cal BP with the `R_Date Function` estimating an age range of 9019–5492 cal BP and `Interval Function` duration of 1315–4657 years.
-Zone T4: Place the start of the `Phase 4` from 6772–4353 and end around 2329– -2001 cal BP with the `R_Date Function` estimating an age range of 6533–502 cal BP and `Interval Function` duration of 0–9144 years.

1.2 OxCal Code associated with Bayesian Model, S5

```
Plot()
 {
  Outlier_Model("General",T(5),U(0,4),"t");
  Sequence("Tequendama")
  {
   Boundary("Start 1");
   Phase("1")
   {
    R_Date("Col 170; GrN-6539",10920,260)
    {
     Outlier("General", 0.05);
    };
    R_Date("Col 167; GrN-6505",10590,90)
    {
     Outlier("General", 0.05);
    };
    R_Date("Col 166; GrN-6270",10730,105)
    {
     Outlier("General", 0.05);
    };
    R_Date("Col 169; GrN-6731",10460,130)
```

```
  {
   Outlier("General", 0.05);
  };
 R_Date("Col 175; GrN-7114",10150,150)
  {
   Outlier("General", 0.05);
  };
 R_Date("Col 168; GrN-7113",10140,100)
  {
   Outlier("General", 0.05);
  };
 R_Date("Col 176/177; GrN-6732",10130,150)
  {
   Outlier("General", 0.05);
  };
 R_Date("Col 165; GrN-6210",10025,95)
  {
   Outlier("General", 0.05);
  };
 R_Date("Col 174; GrN-6730",9900,110)
  {
   Outlier("General", 0.05);
  };
 R_Date("Col 171; GrN-7115",9740,135)
  {
   Outlier("General", 0.05);
  };
 Interval("Duration 1");
 Date("Date 1");
};
Boundary("1_2");
Phase("2")
{
 Interval("Duration 2");
 Date("Date 2");
};
Boundary("2_3");
Phase("3")
{
 R_Date("Col 182; GrN 7477; Ind. 12",7235,35)
  {
   Outlier("General", 0.05);
  };
 R_Date("Col 164; GrN-6729",7090,75)
  {
   Outlier("General", 0.05);
  };
 R_Date("Col 163; GrN-6728",6990,110)
  {
   Outlier("General", 0.05);
  };
```

```
    R_Date("Col 161; GrN-6537",6395,70)
    {
     Outlier("General", 0.05);
    };
    R_Date("Col 181; GrN 7478; Ind. 13",6020,45)
    {
     Outlier("General", 0.05);
    };
    R_Date("Col 180; GrN 7476; Ind. 7",5805,50)
    {
     Outlier("General", 0.05);
    };
    Interval("Duration 3");
    Date("Date 3");
   };
   Boundary("3_4");
   Phase("4")
   {
    R_Date("Col 159; GrN-6536",2225,35)
    {
     Outlier("General", 0.05);
    };
    Difference("Phase 4 Span","4_End","3_4")
    {
     Outlier("General", 0.05);
    };
    Interval("Duration 4");
    Date("Date 4");
   };
   Boundary("4_End");
  };
 };
```

1.3 Notes on OxCal Code associated Bayesian Model, S6

Building upon Bayesian Model #1, this model includes one date from previously published literature[56,57] and 6 new dates from this study. `Rcombine` helps estimate an average or combined value for duplicate samples OxA-43250 and OxA-43251. In the `Outlier Model`, `SSimple` was only used for these duplicate samples, whereas default options have been used for all other samples. Results indicate there is only one major (>60%) outlier in the sequence (sample Col-AAA , laboratory code is unreported), within `Phase 2` (Zone T2). Although the sample is reported to originate from the same stratigraphic contexts that make up Zone T2, this was sourced from a re-excavation of the site and there remains uncertainty. Due to possible stratigraphic uncertainty, boundaries between phases are shared. Model outputs;
-Zone T1: Place the start of the `Phase 1` from 13525–12543 and end around 11051–10435 cal BP with the `R_Date Function` estimating an age range of 12991–10641 cal BP and `Interval Function` duration of 1624–2958 years.

-Zone T2: Place the start of the `Phase 2` from 11051–10435 and end around 10207–7968 cal BP with the `R_Date Function` estimating an age range of 10750–8256 cal BP and `Interval Function` duration of 601–3002 years.
-Zone T3: Place the start of the `Phase 3` from 10207–7968 and end around 6608–5894 cal BP with the `R_Date Function` estimating an age range of 8830–6092 cal BP and `Interval Function` duration of 1437–3701 years.
-Zone T4: Place the start of the `Phase 4` from 6608–5894 and end around 2329– -1478 cal BP with the `R_Date Function` estimating an age range of 6484–816 cal BP and `Interval Function` duration of 3649–7836 years.

1.4 OxCal Code associated with Bayesian Model, S6

```
Plot()
 {
  Outlier_Model("General",T(5),U(0,4),"t");
  Outlier_Model("SSimple",N(0,2),0,"s");
  Sequence("Tequendama")
  {
   Boundary("Start 1");
   Phase("1")
   {
    R_Date("Col 170; GrN-6539",10920,260)
    {
     Outlier("General", 0.05);
    };
    R_Date("Col 167; GrN-6505",10590,90)
    {
     Outlier("General", 0.05);
    };
    R_Date("Col 166; GrN-6270",10730,105)
    {
     Outlier("General", 0.05);
    };
    R_Date("Col 169; GrN-6731",10460,130)
    {
     Outlier("General", 0.05);
    };
    R_Date("Col 175; GrN-7114",10150,150)
    {
     Outlier("General", 0.05);
    };
    R_Date("Col 168; GrN-7113",10140,100)
    {
     Outlier("General", 0.05);
    };
    R_Date("Col 176/177; GrN-6732",10130,150)
    {
     Outlier("General", 0.05);
    };
```

```
 R_Date("Col 165; GrN-6210",10025,95)
 {
  Outlier("General", 0.05);
 };
 R_Date("Col 174; GrN-6730",9900,110)
 {
  Outlier("General", 0.05);
 };
 R_Date("Col 171; GrN-7115",9740,135)
 {
  Outlier("General", 0.05);
 };
 R_Combine("duplicates")
 {
  R_Date("OxA-43251; TEQ-056-03",9490,32)
  {
   Outlier("SSimple", 0.05);
  };
  R_Date("OxA-43350; TEQ-056-03",9487,31)
  {
   Outlier("SSimple", 0.05);
  };
  Outlier("General", 0.05);
 };
 Interval("Duration 1");
 Date("Date 1");
};
Boundary("1_2");
Phase("2")
{
 R_Date("OxA-43306; TEQ-042-02",9026,28)
 {
  Outlier("General", 0.05);
 };
 R_Date("OxA-43307; TEQ-037-03",9287,29)
 {
  Outlier("General", 0.05);
 };
 R_Date("Col-AAA",6080,40)
 {
  Outlier("General", 0.05);
 };
 Interval("Duration 2");
 Date("Date 2");
};
Boundary("2_3");
Phase("3")
{
 R_Date("Col 182; GrN 7477",7235,35)
 {
  Outlier("General", 0.05);
```

```
    };
    R_Date("Col 164; GrN-6729",7090,75)
    {
     Outlier("General", 0.05);
    };
    R_Date("Col 163; GrN-6728",6990,110)
    {
     Outlier("General", 0.05);
    };
    R_Date("Col 161; GrN-6537",6395,70)
    {
     Outlier("General", 0.05);
    };
    R_Date("Col 181; GrN 7478",6020,45)
    {
     Outlier("General", 0.05);
    };
    R_Date("Col 180; GrN 7476",5805,50)
    {
     Outlier("General", 0.05);
    };
    R_Date("OxA-43304; TEQ-018-02",5773,22)
    {
     Outlier("General", 0.05);
    };
    Interval("Duration 3");
    Date("Date 3");
   };
   Boundary("3_4");
   Phase("4")
   {
    R_Date("OxA-43305; TEQ-034-01",5081,20)
    {
     Outlier("General", 0.05);
    };
    R_Date("Col 159; GrN-6536",2225,35)
    {
     Outlier("General", 0.05);
    };
    Interval("Duration Phase 4");
    Date("Date 4");
   };
   Boundary("4_End");
  };
 };
```

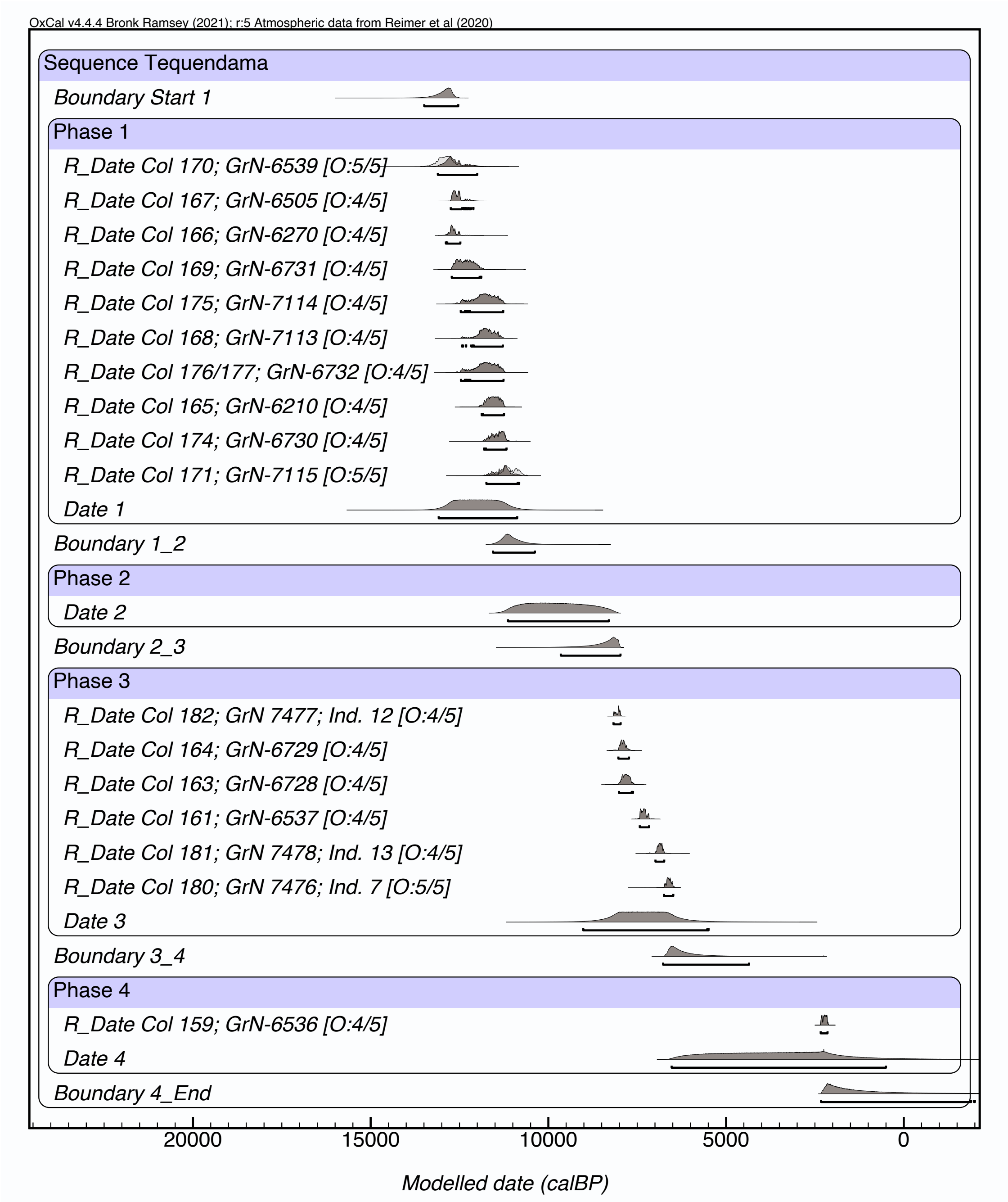

FIGURE S5: OxCal Bayesian modelling of the radiocarbon dates available for the archeological site Tequendama, specifically those published prior to this study, related to Table 2, Table S3 and STAR Methods. Calibrated dates across all zones of occupation (T1–T4) include those exclusively from the original excavation and publication.[39] The zones of occupation have been modeled as uniform phases using OxCal4.4[113, 114] and reported in cal BP. Brackets beneath each age estimate show 95.4% CI. Outlier analysis output is noted as ‘O:posterior probability/prior probability’.[161]

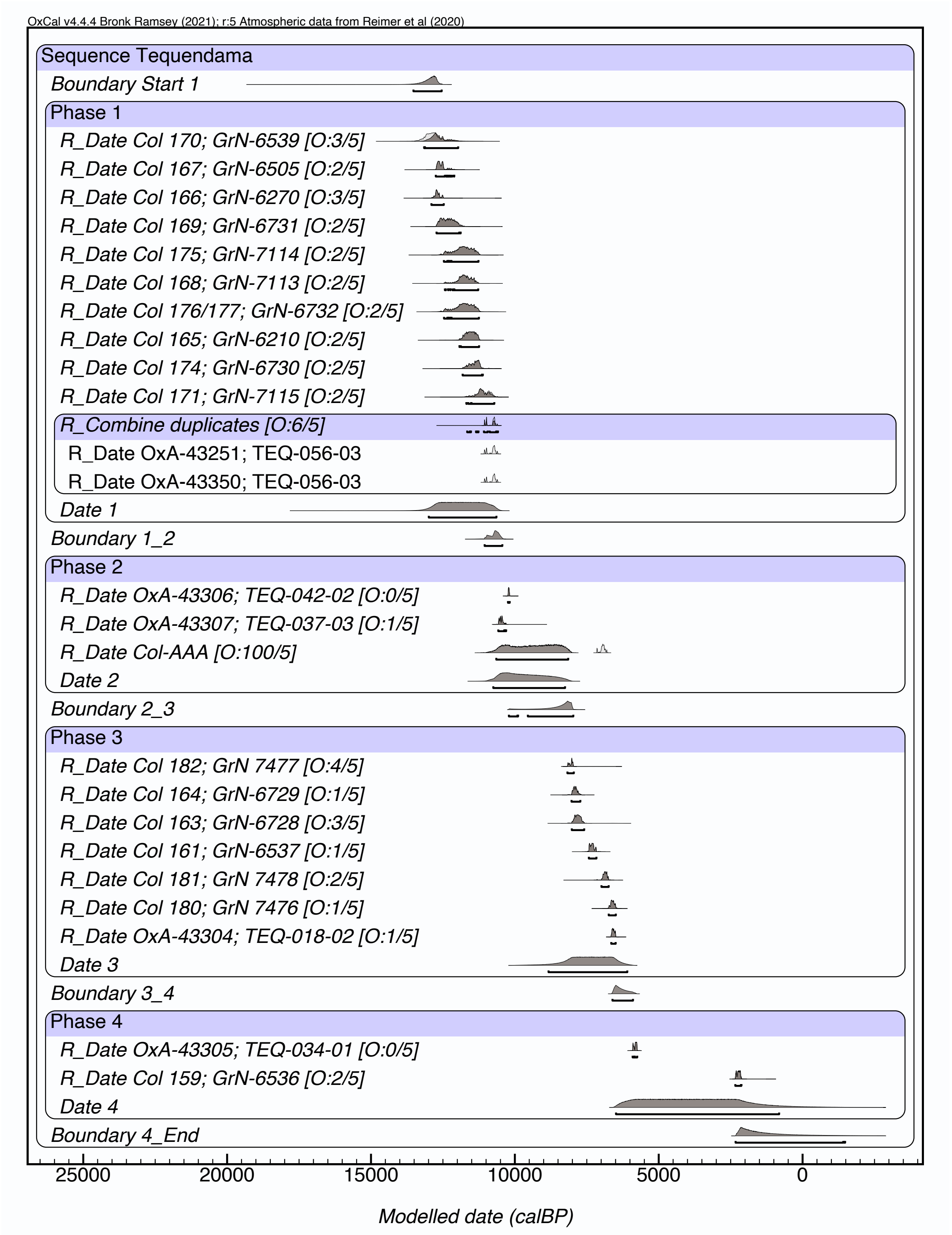

FIGURE S6: OxCal Bayesian modelling of the radiocarbon dates available for the archeological site Tequendama and those provided by this study, related to Table 2, Table S3 and STAR Methods. Calibrated radiocarbon dates across all zones of occupation (T1–T4) include those from the original excavation and publication[39], a brief site reexamination by other authors[56,57] and resulting chronological data (n=6) presented in this study. The zones of occupation have been modeled as uniform phases using OxCal4.4[113, 114] and reported in cal BP. Brackets beneath each age estimate show 95.4% CI. Outlier analysis output is noted as 'O:posterior probability/prior probability'. [161]

S1 REFERENCES

134. Gómez Tapias, J., Núñez–Tello, A., Mateus–Zabala, D., Alcárcel–Gutiérrez, F.A., Lasso–Muñoz, R.M., Marín–Rincón, E., and and Marroquín–Gómez, M.P. (2020). Physiographic and Geological Setting of the Colombian Territory. In The Geology of Colombia Publicaciones Geológicas Especiales., J. Gómez and D. Mateus–Zabala, eds. (Servicio Geológico Colombiano), pp. 1–34. doi: 10.32685/pub.esp.35.2019.01.

135. Correal Urrego, G. (1990). EVIDENCIAS CULTURALES DURANTE EL PLEISTOCENO Y HOLOCENO DE COLOMBIA. Revista de Arqueología Americana, 69–89.

136. Olson, D.M., Dinerstein, E., Wikramanayake, E.D., Burgess, N.D., Powell, G.V.N., Underwood, E.C., Itoua, I., Strand, H.E., Morrison, J.C., Loucks, C.J., et al. (2001). Terrestrial Ecoregions of the World: A New Map of Life on EarthA new global map of terrestrial ecoregions provides an innovative tool for conserving biodiversity. Bioscience *51*, 933–938. doi: 10.1641/0006-3568(2001)051[0933:TEOTWA]2.0.CO;2.

137. de Vivo, M., and Carmignotto, A.P. (2004). Holocene Vegetation Change and the Mammal Faunas of South America and Africa. J. Biogeogr. *31*, 943–957.

138. Gómez, J., & Pinilla–Pachon, A.O. ed. (2020). The Geology of Colombia, Quaternary (Servicio Geológico Colombiano) doi: 10.32685/pub.esp.38.2019.

139. Antonelli, A., Zizka, A., Carvalho, F.A., Scharn, R., Bacon, C.D., Silvestro, D., and Condamine, F.L. (2018). Amazonia is the primary source of Neotropical biodiversity. Proc. Natl. Acad. Sci. U. S. A. *115*, 6034–6039. doi: 10.1073/pnas.1713819115.

140. van der Hammen, T. (1974). The Pleistocene Changes of Vegetation and Climate in Tropical South America.

141. Hoorn, C., Wesselingh, F.P., ter Steege, H., Bermudez, M.A., Mora, A., Sevink, J., Sanmartín, I., Sanchez-Meseguer, A., Anderson, C.L., Figueiredo, J.P., et al. (2010). Amazonia through time: Andean uplift, climate change, landscape evolution, and biodiversity. Science *330*, 927–931. doi: 10.1126/science.1194585.

142. Hooghiemstra, H., and Van der Hammen, T. (2004). Quaternary Ice-Age dynamics in the Colombian Andes: developing an understanding of our legacy. Philos. Trans. R. Soc. Lond. B Biol. Sci. *359*, 173–181. doi: 10.1098/rstb.2003.1420.

143. van Der Hammen, T., and Hooghiemstra, H. (1995). The EL abra stadial, a younger dryas equivalent in Colombia. Quat. Sci. Rev. *14*, 841–851. doi: 10.1016/0277-3791(95)00066-6.

144. Martínez-Polanco, M.F. (2016). El Cuy (Cavia Sp.), Un Recurso Alimenticio Clave en Aguazuque, Un Sitio Arqueológico de la Sabana de Bogotá, Colombia. Latin American Antiquity *27*, 512–526. doi: 10.7183/1045-6635.27.4.512.

145. Tejada, J.V., Flynn, J.J., MacPhee, R., O'Connell, T.C., Cerling, T.E., Bermudez, L., Capuñay, C., Wallsgrove, N., and Popp, B.N. (2021). Isotope data from amino acids indicate Darwin's ground sloth was not an herbivore. Sci. Rep. *11*, 18944. doi: 10.1038/s41598-021-97996-9.

146. Thompson, L.G., Davis, M.E., Mosley-Thompson, E., Sowers, T.A., Henderson, K.A., Zagorodnov, V.S., Lin, P., Mikhalenko, V.N., Campen, R.K., Bolzan, J.F., et al. (1998). A 25,000-year tropical climate history from bolivian ice cores. Science *282*, 1858–1864. doi: 10.1126/science.282.5395.1858.

147. Seltzer, G., Rodbell, D., and Burns, S. (2000). Isotopic evidence for late Quaternary climatic change in tropical South America. Geology *28*, 35–38. doi: 10.1130/0091-7613(2000)28<35:IEFLQC>2.0.CO;2.

148. Sponheimer, M., and Lee-Thorp, J.A. (1999). Oxygen isotopes in enamel carbonate and their ecological significance. J. Archaeol. Sci. *26*, 723–728. doi: 10.1006/jasc.1998.0388.

149. Pederzani, S., and Britton, K. (2019). Oxygen isotopes in bioarchaeology: Principles and applications, challenges and opportunities. Earth-Sci. Rev. *188*, 77–107. doi: 10.1016/j.earscirev.2018.11.005.

150. Tangarife-Escobar, A., Koeniger, P., López-Moreno, J.I., Botía, S., and Ceballos-Liévano, J.L. (2023). Spatiotemporal variability of stable isotopes in precipitation and stream water in a high elevation tropical catchment in the Central Andes of Colombia. Hydrol. Process. *37*. doi: 10.1002/hyp.14873.

151. Gonfiantini, R., Roche, M.-A., Olivry, J.-C., Fontes, J.-C., and Zuppi, G.M. (2001). The altitude effect on the isotopic composition of tropical rains. Chem. Geol. *181*, 147–167. doi: 10.1016/S0009-2541(01)00279-0.

152. Saylor, J.E., Mora, A., Horton, B.K., and Nie, J. (2009). Controls on the isotopic composition of surface water and precipitation in the Northern Andes, Colombian Eastern Cordillera. Geochim. Cosmochim. Acta *73*, 6999–7018. doi: 10.1016/j.gca.2009.08.030.

153. Boutton, T.W., Lynott, M.J., and Bumsted, M.P. (1991). Stable carbon isotopes and the study of prehistoric human diet. Crit. Rev. Food Sci. Nutr. *30*, 373–385. doi: 10.1080/10408399109527548.

154. Reynard, B., and Balter, V. (2014). Trace Elements and their Isotopes in Bones and Teeth: Diet, Environments, Diagenesis, and Dating of Archeological and Paleontological Samples. Palaeogeogr. Palaeoclimatol. Palaeoecol. *416*, 4–16. doi: 10.1016/j.palaeo.2014.07.038.

155. Turner Tomaszewicz, C.N., Seminoff, J.A., Avens, L., and Kurle, C.M. (2016). Methods for sampling sequential annual bone growth layers for stable isotope analysis. Methods Ecol. Evol. *7*, 556–564. doi: 10.1111/2041-210x.12522.

156. van der Merwe, N.J., and Vogel, J.C. (1978). 13C content of human collagen as a measure of prehistoric diet in woodland North America. Nature *276*, 815–816. doi: 10.1038/276815a0.

157. Katzenberg, M.A., and Waters-Rist, A.L. (2018). Stable isotope analysis. In Biological Anthropology of the Human Skeleton, M. Anne Katzenberg and Anne L. Grauer, ed. (John Wiley & Sons, Inc.), pp. 467–504. doi: 10.1002/9781119151647.ch14.

158. Caut, S., Angulo, E., and Courchamp, F. (2009). Variation in discrimination factors (Δ^{15}N and Δ^{13}C): the effect of diet isotopic values and applications for diet reconstruction. J. Appl. Ecol. *46*, 443–453. https://doi.org/10.1111/j.1365-2664.2009.01620.x.

159. Fernandes, R., Millard, A.R., Brabec, M., Nadeau, M.-J., and Grootes, P. (2014). Food reconstruction using isotopic transferred signals (FRUITS): a Bayesian model for diet reconstruction. PLoS One *9*, e87436. doi: 10.1371/journal.pone.0087436.

160. O'Connell, T.C., Kneale, C.J., Tasevska, N., and Kuhnle, G.G.C. (2012). The diet-body offset in human nitrogen isotopic values: a controlled dietary study. Am. J. Phys. Anthropol. *149*, 426–434. doi: 10.1002/ajpa.22140.

161. Bronk Ramsey, C. (2009). Dealing with outliers and offsets in radiocarbon dating. *Radiocarbon 51*, 1023–1045. doi: 10.1017/s0033822200034093.
